# An ancient whole-genome duplication in barnacles contributes to their diversification and intertidal sessile life adaptation

**DOI:** 10.1016/j.jare.2023.09.015

**Published:** 2023-09-20

**Authors:** Jianbo Yuan, Xiaojun Zhang, Xiaoxi Zhang, Yamin Sun, Chengzhang Liu, Shihao Li, Yang Yu, Chengsong Zhang, Songjun Jin, Min Wang, Jianhai Xiang, Fuhua Li

**Affiliations:** a CAS and Shandong Province Key Laboratory of Experimental Marine Biology, Center for Ocean Mega-Science, Institute of Oceanology, Chinese Academy of Sciences, Qingdao 266071, China; b Laboratory for Marine Biology and Biotechnology, Qingdao National Laboratory for Marine Science and Technology, Qingdao 266237, China; c Research Center for Functional Genomics and Biochip, Tianjin 300457, China; d TEDA Institute of Biological Sciences and Biotechnology, Nankai University, Tianjin 300457, China; e Key Laboratory of Molecular Microbiology and Technology, Ministry of Education, Nankai University, Tianjin 300071, China

**Keywords:** Barnacle genome, Whole-genome duplication, Comparative genomics, Intertidal adaptation, Transcriptome analysis

## Abstract

**Introduction:**

Whole-genome duplication (WGD) is one of the most sudden and dramatic events rarely reported in invertebrates, but its occurrence can lead to physiological, morphological, and behavioral diversification. WGD has also never been reported in barnacles, which is one of the most unique groups of crustaceans with extremely speciallized morphology (calcareous shells) and habits (intertidal sessile lifestyle).

**Objectives:**

To investigate whether WGD has occurred in barnacles and examine its potential role in driving the adaptive evolution and diversification of barnacles.

**Methods:**

Based on a newly sequenced and assembled chromosome-level barnacle genome, a novel WGD event has been identified in barnacles through a comprehensive analysis of interchromosomal synteny, the Hox gene cluster, and synonymous substitution distribution.

**Results:**

We provide ample evidences for WGD in the barnacle genomes. Comparative genomic analysis indicates that this WGD event predates the divergence of Thoracicalcarea, occurring more than 247 million years ago. The retained ohnologs from the WGD are primarily enriched in various pathways related to environmental information processing, shedding light on the adaptive evolution and diversification of intertidal sessile lifestyle. In addition, transcriptomic analyses show that most of these ohnologs were differentially expressed following the ebb of tide. And the cytochrome P450 ohnologs with differential expression patterns are subject to subfunctionalization and/or neofunctionalization for intertidal adaptation. Besides WGD, parallel evolution underlying intertidal adaptation has also occurred in barnacles.

**Conclusion:**

This study revealed an ancient WGD event in the barnacle genomes, which is potentially associated with the origin and diversification of thoracican barnacles, and may have contributed to the adaptive evolution of their intertidal sessile lifestyle.

## Highlights

• We sequenced and assembled a chromosome-level barnacle genome.
• A WGD event was identified in barnacles for the first time.
• This WGD event is more ancient than the divergence of Thoracicalcarea.
• Ohnologs were subject to sub- or neo-functionalization for intertidal adaptation.
• The ancient WGD event may associate with the origin and diversification of thoracican barnacles.

## Graphical abstract

**Figure:**
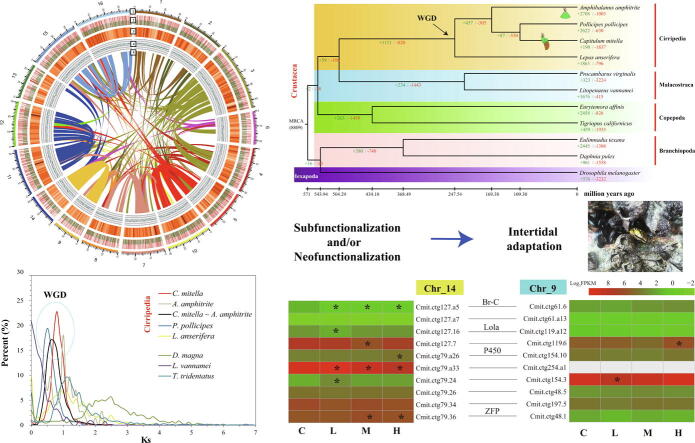

## Introduction

Whole-genome duplication (WGD) is characterized by doubling the entire genetic repertoire of an organism, which is particularly important for generating new genes and further leading to the divergence and formation of new species over time [1]. WGD is arguably the most sudden and massive change that a genome can experience in a single evolutionary event [2]. The occurrence of WGD has been widely reported in plants and vertebrates [3], [4], [5], whereas in invertebrates, only a few taxa, such as bdelloid rotifers, horseshoe crabs, and house spiders, have been demonstrated to undergo WGD [2], [6], [7]. WGD can make profound impacts on the diversity of organisms in terms of genetics, physiology, morphology, behavior, and ecology, for example, leading to the diversification of crop domestication, fish pigmentation, and migratory behavior in Salmonidae [8], [9], [10], [11]. More importantly, in some cases, WGDs have been linked to the origin of some unique groups of organisms, such as angiosperms and teleosts [12], [13].

Barnacles are a type of crustacean belonging to the subclass Cirripedia (Thecostraca), and hence are closely related to other marine crustaceans, such as shrimp and crabs. However, barnacles have a number of morphological characteristics and habits that are distinct from those of other crustaceans, including a calcareous exoskeleton, a sessile lifestyle, and adaptation to an intertidal habitat (Supplementary Fig. S1) [14]. Unlike other crustaceans that are free-swimming throughout their lives, barnacles cannot move after their larval settlement. They lack an abdomen, and some parasitic barnacles even have a loss of segmentation in adulthood [15]. Their bodies are placed upside down after attachment, and they feed via feather-like appendages that have been modified into cirri, which are used for plankton filtration and respiration [16], [17].

Living in the intertidal zone is full of challenges for organisms because they must withstand fluctuations in numerous environmental stressors, such as temperature (thermal stress in summer), salinity, hypoxia, air exposure, pathogens, and the ebb and flow of the tide [18], [19]. However, unlike other intertidal crustaceans (e.g., crabs and amphipods) that can hide in the sand or under rocks to protect themselves, barnacles must endure these stressful conditions because they are sessile creatures. Thus, the intertidal zone is much more stressful for barnacles than for other crustaceans. In summary, barnacles show an apparent diversification in morphological and ecological phenotypes compared to other crustaceans, which raises our interest in identifying what has driven the origin and dramatic changes in their phenotypes, as well as whether they are related to the WGD.

Except for the oriental river prawn *Macrobrachium nipponense*, WGD events have not been reported in barnacles and other crustaceans [20]. However, it has been reported that a diploidization event may have occurred during the evolution of a single genus of parasitic barnacles [21]. Also, a conserved developmental gene, *engrailed*, was reported to have been duplicated during the evolution of barnacles [15]. Thus, WGD may have occurred in barnacles, and sequencing a high-quality chromosome-level genome will help clarify this issue.

There are four barnacle species whose genomes have been reported, including one acorn barnacle, *Amphibalanus amphitrite*, and three stalked barnacles, *Capitulum mitella*, *Pollicipes pollicipes*, and *Lepas anserifera* [22], [23], [24], [25]. However, WGD events have not been reported in these studies. Although interchromosomal synteny analysis has been performed on the *C. mitella* genome, limited syntenic blocks were identified, resulting in the failure to detect WGD events [22]. In this study, we reported another chromosome-level genome of *C. mitella*, and conducted transcriptomics and metabolomics sequencing and analyses on this species to investigate its unique adaptive mechanisms to intertidal life. Interestingly, for the first time, we found strong evidence of a WGD event that occurred in barnacles. Further comparative analyses indicated that this WGD event may have contributed to the diversification of barnacles and the adaptive evolution of the intertidal sessile lifestyle.

## Material and methods

### Ethics statement

All experiments involving animals were conducted according to the ethical policies and procedures approved by the ethics committee of the Animal Ethics Committee at the Institute of Oceanology, Chinese Academy of Sciences (Approval no. 2020(37)).

### Genome sequencing

The samples of *C. mitella* were collected from the intertidal zone of Zhoushan, China (122.43 E, 29.95 N). Genomic DNA was extracted from the whole body of a single stalked barnacle using the TIANamp Marine Animals DNA Kit according to the manufacturer's instructions (Tiangen, Beijing, China). Then, the DNA was used for genome sequencing on the HiSeq X-TEN sequencing platform (Illumina, San Diego, CA, USA) and the PacBio Sequel sequencing platform (Pacific Biosciences, Menlo Park, CA, USA). In addition, a Hi-C library was constructed and sequenced to anchor the contigs to the chromosomes. Detailed methods of genomic library construction and sequencing are described in Supplementary Note 1.

### Genome size estimation

The genome size of *C. mitella* was estimated by the distribution of K-mer (substrings of length K in a given sequencing read) frequency. K-mer frequencies were calculated using Jellyfish based on the cleaned reads from the Illumina sequencing data [26], which is widely used for the estimation of genome size and repeat content. Then, genome size and repeat content was estimated using the software GenomeScope 2.0 (Supplementary Fig. S2) [27].

### Genome assembly

The *C. mitella* genome was *de novo* assembled into contigs using WTDBG2 [28] and then assembled into chromosome-level using Juicer [29]. The 3D-DNA pipeline (version 180419) was used to assign the order and orientation of each group [30]. The genome assembly quality was also evaluated using Illumina sequencing reads and RNA-seq data coverage, as well as the benchmarking universal single-copy ortholog (BUSCO) analysis. Detailed methods of genome assembly are described in Supplementary Note 1.

### Genome annotation

RepeatModeler and RepeatMasker were used for *de novo* annotation of transposable elements (TEs) [31]. A combination of homology-based prediction, *ab initio* prediction, and transcriptome-based prediction was utilized for protein-coding gene annotation. Functional annotations of the predicted genes were conducted using BLASTP against the NCBI-NR, SwissProt, and KOG databases with an E-value of 1E^-05^. Functional enrichment analysis was conducted on a subset of genes according to their GO and KEGG classifications. Detailed methods of genome annotation and enrichment analyses are described in Supplementary Note 2.

### Gene family and phylogenetic analyses

Gene family clustering was performed on 11 arthropods using OrthoFinder. The protein-coding genes of 11 arthropods were collected from NCBI genome database and used for gene family analysis, including *C. mitella* (this study, PRJNA753937), *A. amphitrite* (PRJNA751628), *P. pollicipes* (PRJNA624368), *L. anserifera* (PRJNA678024), *Procambarus virginalis* (PRJNA356499), *Litopenaeus vannamei* (PRJNA438564), *Eurytemora affinis* (PRJNA423276), *Tigriopus californicus* (PRJNA237968), *Eulimnadia texana* (PRJNA352082), *Daphnia pulex* (PRJNA794129), and *Drosophila melanogaster* (PRJNA559813). The expansion and contraction of gene families were quantified using CAFÉ [32]. The conserved single-copy orthologous genes were used for phylogenetic tree construction via the maximum likelihood method. Divergence times were estimated through a combined analysis with the programs of r8s and RAxML [33]. Detailed methods for phylogenetic analysis are described in Supplementary Note 3.

### Whole-genome duplication analysis

To infer the WGD events in the barnacles, we performed a series of analyses on the *C. mitella* genome, including interchromosomal synteny block identification, Hox gene cluster comparison, conserved gene cluster location, and synonymous substitution (Ks) distribution analysis. To identify the syntenic blocks, an all-against-all BLASTP method (E value < 1e-05) was used to detect the paralogous genes in the *C. mitella* genome and the genomes of *A. amphitrite*, *P. pollicipe*, *Daphnia magna*, *T. tridentatus* and *L. vannamei*. In addition, the orthologous genes between *C. mitella* and *A. amphitrite* were identified using the same BLASTP method. Synteny blocks with at least five collinear orthologous genes were detected using the MCScanX software [34]. Genes were further classified using a duplicate gene-classifier in MCScanX. Since limited syntenic blocks were identified in the *P. pollicipe* genome, the paralogous chromosomes were identified by calculating the interchromosomal best-hit paralogs in the genome.

The Ks values of the intraspecific and interspecific blocks were calculated using the HKY model [2], [35]. The Hox gene cluster was identified in 10 arthropod genomes, including two species with WGD, *T. tridentatus* and *Parasteatoda tepidariorum*.

### Transcriptome sequencing and analyses

A transcriptome of *C. mitella* was sequenced to perform transcriptome-based gene prediction. The whole body of *C. mitella* individuals was collected and stored at − 80 °C. To investigate the mechanisms of intertidal adaptation, transcriptome sequencing and comparative analyses of *C. mitella* were performed. Individuals of *C. mitella* were sampled *in situ* from a large population of intertidal rocky pools in Zhoushan, Zhejiang, China, in October with air temperatures of 18 ∼ 24 ℃. The time of air exposure is proportionate to the altitude of the attached rocks under natural conditions. In this study, individuals attached at high (H, ∼4 m above the low-tide line), medium (M, ∼2 m above the low-tide line), and low (L, ∼0.5 m above the low-tide line) elevations were sampled, and individuals exposed to seawater served as controls (C). All samples were immediately frozen in liquid nitrogen.

RNA isolation and transcriptome sequencing were conducted according to standard protocols. According to the standard manufacturer’s protocol, the total RNA was isolated and purified from the samples using TRIzol extraction reagent (Thermo Fisher Scientific, USA). RNA quality and concentration were determined by 1% agarose gel electrophoresis, and RNA concentration was assessed using a Nanodrop 2000 spectrophotometer (Thermo Fisher Scientific, USA). Transcriptome libraries were prepared according to the instructions of the TruSeq RNA Library Prep Kit (Illumina, San Diego, USA), and then sequenced on the Illumina HiSeq 2500 platform. Based on the transcriptome data, the TopHat v1.2.1 package was used to map the transcriptome reads to the barnacle genomes [36]. Then, the fragments per kilobase of transcript per million fragments mapped (FPKM) values were calculated using Cufflinks v2.2.1. In addition, differential gene expression analysis was conducted using edgeR v3.10 [37].

### Metabolome sequencing and analyses

In order to investigate the metabolites involved in intertidal adaptation, we performed metabolome sequencing and analyses of *C. mitella*. The samples (C and M) collected from Zhoushan were used for metabolome sequencing. Each sample (50 mg) was homogenized with 1000 µl of ice-cold methanol/water (70%, v/v). Cold steel balls were added to the mixture, and then homogenized for 3 min at 30 Hz. The mixture was vortexed for 1 min and then centrifuged at 12,000 rpm for 10 min at 4 ℃. The collected supernatant was used for LC-MS/MS analysis using an LC-ESI-MS/MS system (UPLC, Shim-pack UFLC SHIMADZU CBM A system; MS, QTRAP® System). LIT and triple quadrupole (QQQ) scans were acquired on a triple quadrupole-linear ion trap mass spectrometer (QTRAP), The QTRAP® LC-MS/MS System was equipped with an ESI Turbo Ion-Spray interface that operated in positive and negative ion mode and controlled by Analyst® 1.6.3 software (https://sciex.com/products/software/analyst-software). Instrument tuning and mass calibration were performed with 10 and 100 μmol/L polypropylene glycol solutions in the QQQ and LIT modes, respectively.

Orthogonal partial least squares discriminant analysis (OPLS-DA) was used to identify the differential metabolites [38]. Two criteria were used to assess the significance of differential metabolite levels. One was the fold change of the variable, and the other was the variable importance in projection (VIP) value. A metabolite was considered to be differentially regulated when the fold change was ≥ 2 or ≤ 0.5, and VIP was ≥ 1. The VIP value was calculated using the OPLSR.Anal function of the MetaboAnalystR R package [39]. Prior to OPLS-DA, the data were log transformed (log2) and mean centered.

## Results

### Genome assembly, annotation, and comparative analysis

Based on the genome sequencing data in the form of 49 Gb of Illumina paired-end reads (96×), 57 Gb of PacBio long reads (112×), and 142 Gb of Hi-C sequencing data (277×, Supplementary Table S1), a high-quality chromosome-level genome of the stalked barnacle, *C. mitella*, was assembled (Supplementary Note 1). For the purpose of identifying WGD events, a haploid assembler (WTDBG2) was used to generate the haplotype assembly of *C. mitella* [28]. The final assembled genome size was 512.06 Mb with contig and scaffold N50 lengths of 3.22 Mb and 30.73 Mb, respectively (Supplementary Table S2, Fig. S2, S3). This assembly showed high continuity and completeness (Supplementary Tables S3-S5, Fig. S4), comparable to the genome assembled in the previous study [22]. However, when compared to the genome assembled by Chen (https://ngdc.cncb.ac.cn/gwh/Assembly/21261/show) (478 Mb and 8080 protein-coding genes with BUSCO coverage of 67%), we found that the genome assembled in this study has superiority in assembly size (512 Mb) and annotated genes (13,364 genes with BUSCO coverage of 93%)(Supplementary Note 1). Furthermore, our assembly identified significantly more interchromosomal syntenic blocks than the previous assembly, which provided important clues for WGD, as described in the sections below.

**Fig. 1:**
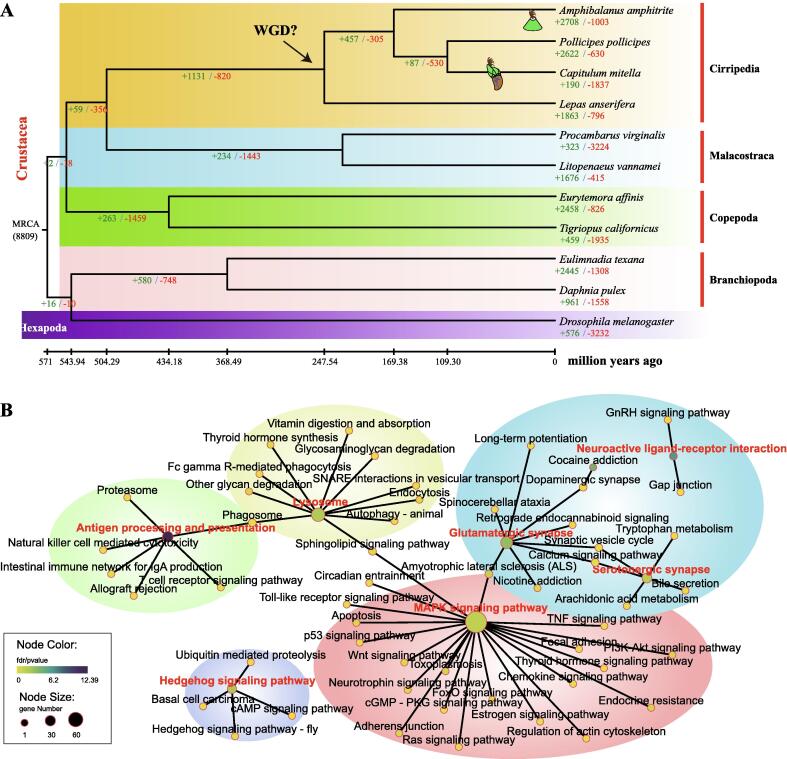
**Comparative genomic analyses of barnacles and their relatives. (A)** The phylogenetic placement of barnacles in the arthropod phylogenetic tree. The numbers on the branches indicate the number of gene gains (+) or losses (-). The estimated divergence times are displayed below the phylogenetic tree. **(B)** The network plot of the functional enrichment of the expanded gene families. Each node in the plot indicates a KEGG pathway, and the color indicates the enrichment factor (fdr/*p* value). The node size indicates the number of genes involved in the corresponding pathway. Abbreviations: WGD, whole-genome duplication; MRCA, most recent common ancestor.

Based on the present genome assembly, 171 Mb of repetitive sequences (33.44%) and 13,364 protein-coding genes were annotated (Supplementary Tables S6, S7, Fig. S5, S6). The annotated genes showed high completeness (BUSCO coverage of 93.15%). Based on 127 single-copy genes, a high consensus phylogenetic tree of the four barnacles (*A. amphitrite*, *C. mitella*, *P. pollicipes*, and *L. anserifera*) and their arthropod relatives was constructed (Supplementary Note 3). Estimates of divergence times suggested that Cirripedia diverged from other crustaceans approximately 504 million years ago (mya), close to the timing of the Early Cambrian (Fig. 1A) [40]. The divergence of the four barnacle species was estimated to have occurred approximately 247 mya, just after the Permian-Triassic mass extinction event (∼252 mya).

Comparative genomic analysis revealed a large number of uniquely expanded gene families in Cirripedia (1,131 gene families) (Fig. 1A, Supplementary Note 4). These gene families were primarily enriched in the MAPK and Hedgehog signaling pathways, as well as many pathways related to nerve transduction (e.g., neuroactive ligand-receptor interaction, glutamatergic synapse, and serotonergic synapse) (Fig. 1B; Supplementary Fig. S7). The expansion of these gene families may reflect the adaptive evolution of barnacles to a sessile lifestyle and to stressful intertidal zones.

### Whole-genome duplication in the lineage of barnacles

**Fig. 2:**
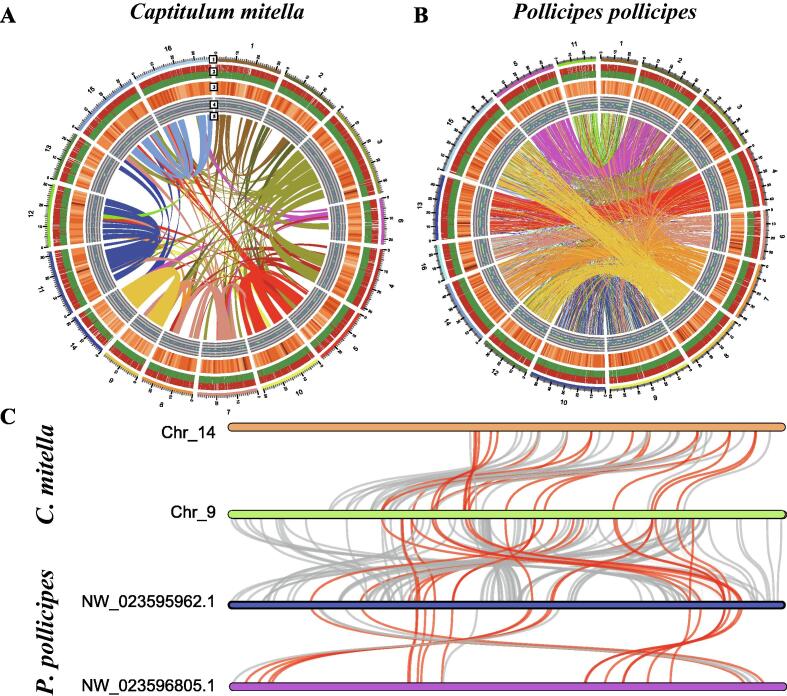
**The interchromosomal synteny of *C. mitella* and *P. pollicipes*. (A)** The interchromosomal synteny of *C. mitella***. (B)** The interchromosomal synteny of *P. pollicipes*. A schematic representation of the genomic characteristics of the two barnacle genomes including (from the outer circle to inner circle): Track 1, 16 chromosomes of the genome; Track 2, protein-coding genes present on the scaffolds, where red represents genes on the forward strand and green represents genes on the reverse strand; Track 3, distribution of the gene density with sliding windows of 1 Mb, where a higher density is indicated by darker red; Track 4, distribution of the GC content in the genome; Track 5, schematic representation of the interchromosomal synteny of the two barnacle genomes, where the arc line indicates the major interchromosomal syntenic blocks in the *C. mitella* genome and the pairwise best hit homologous genes in the *P. pollicipes* genome. **(C)** The conserved synteny blocks between *C. mitella* and *P. pollicipes*. The red lines indicate the ohnologs of the four chromosomes (two paralogous chromosomes originating from the WGD event for *C. mitella* and *P. pollicipes* each). The gray lines indicate the ohnologs of the two paralogous/orthologous chromosomes that were not shared with the other two chromosomes.

To identify a WGD event in barnacles, we performed comprehensive analyses on the genomes of *C. mitella* and three other barnacles (*A. amphitrite*, *P. pollicipes*, and *L. anserifera*). Firstly, a whole-genome synteny analysis was performed on the *C. mitella* genome, and 169 syntenic blocks covering at least five ordered paralogous genes were detected, which was considerably more than those identified in the previous study (10 syntenic blocks) [22], and was comparable to the findings of the horseshoe crab *Tachypleus tridentatus* (320 syntenic blocks), a species that has undergone three rounds of WGD [41], [42]. As expected, the amino acid sequences of these syntenic genes showed relatively low identity (41.58%). These syntenic blocks were not very compact that with occasional insertions of other genes, indicating that these paralogous genes stemmed from an ancient WGD event rather than allelic chromosomes. In addition, these syntenic blocks of paralogous genes were shared between pairwise ohnologous chromosomes (e.g., Chr11 and Chr12) rather than randomly distributed (Fig. 2A), which is similar to the pattern found in the horseshoe crab, but different from that of *M. nipponense* (randomly distributed), a crustacean that is assumed to have undergone WGD [20]. This pattern is consistent with a WGD event rather than independent gene duplication events [41], [42].

Interchromosomal synteny has also been investigated in other barnacle genomes. However, syntenic analysis was not performed on the genomes of *A. amphitrite* and *L. anserifera* due to the lack of a chromosome-level genome for these two species. In the *P. pollicipes* genome, only 28 interchromosomal syntenic blocks were identified, whereas a paralogous chromosomal relationship was apparently observed when examining the distributions of the pairwise best-hit homologous genes (Fig. 2B). This result indicated that the *P. pollicipes* genome had also undergone WGD. If the genome continuity could be improved (the contig N50 length of the *P. pollicipes* genome was 660.8 Kb), more interchromosomal syntenic blocks might be identified [24].

Among the 28 interchromosomal syntenic blocks of *P. pollicipes*, many of them (13 blocks) were also found in the syntenic blocks of *C. mitella* (Fig. 2C). The ohnologs (paralogous genes derived from the WGD) in these blocks were distributed synteny across the four chromosomes of the two barnacle genomes, each with two paralogous chromosomes. Thus, the highly conserved relationships of these shared syntenic blocks indicated that the WGD event may have occurred in the common ancestor of these two barnacle species, and some syntenic ohnologs were retained after the divergence of the two species.

**Fig. 3:**
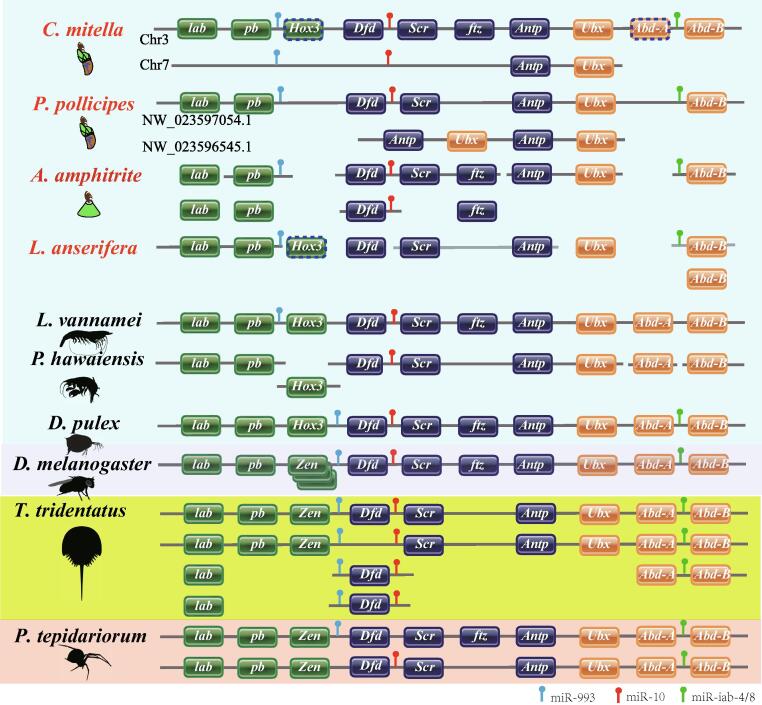
**The Hox gene cluster of ten arthropods.** The boxes linked with a straight line indicate the ordered genes located on a single scaffold or pseudochromosome. The boxes with dotted lines indicate pseudogenes that could not be transcribed or translated. The images of various species were downloaded from the free silhouette images of PHYLOPIC (https://phylopic.org/).

Secondly, as expected from our suggestion of WGD, there were significantly more duplicated BUSCOs in the barnacle genomes (25.66% on average) than in other crustacean genomes (8.62% on average, *P* < 0.05; Supplementary Fig. S4). To provide further insights into the nature of the genome at the gene level after the WGD event, we analyzed a number of conserved gene clusters in barnacles, which were syntenically distributed and generally presented as single-copies in non-WGD genomes (Supplementary Fig. S8). A representative cluster is the Hox gene cluster (Fig. 3), which has been widely used to detect WGD events [2], [6], [7]. Ten conserved syntenic Hox genes and four miRNAs (miR-993, miR-10, and miR-iab-4/8) were present in the four barnacle genomes, although *Hox3* and *Abd-A* were absent (Fig. 3). The absence of *Abd-A* has been associated with the impaired abdominal development in barnacles [43]. However, an incomplete segment of *Abd-A*, located between *Ubx* and *Abd-B*, was identified in the *C. mitella* genome, suggesting the nonfunctionalization of this gene. In addition to the intact Hox gene cluster on Chr3, an additional small cluster consisting of *Antp*, *Ubx*, and two miRNAs (miR-993 and miR-10) was identified on Chr7 of *C. mitella* (Fig. 3), which may have arisen during the WGD event. The additional copies of *Antp* and *Ubx* were also present in the *P. pollicipes* genome, suggesting that they originated from the ancestral genome of these two barnacles (*C. mitella* and *P. pollicipes*). Although no additional copies of *Antp* and *Ubx* were detected in the genomes of *A. amphitrite* and *L. anserifera*, additional copies of other Hox genes (*lab*, *pb*, *Dfd*, *ftz*, and *Abd-B*) were identified in these two genomes (Fig. 3).

In the *C. mitella* genome, relics of the WGD were found in some of the other conserved genes (such as *engrailed*) and gene clusters, but many of them had lost their second copies, similar to the Hox gene cluster (Supplementary Fig. S8). These findings suggest that the WGD event may have occurred a long time ago.

**Fig. 4:**
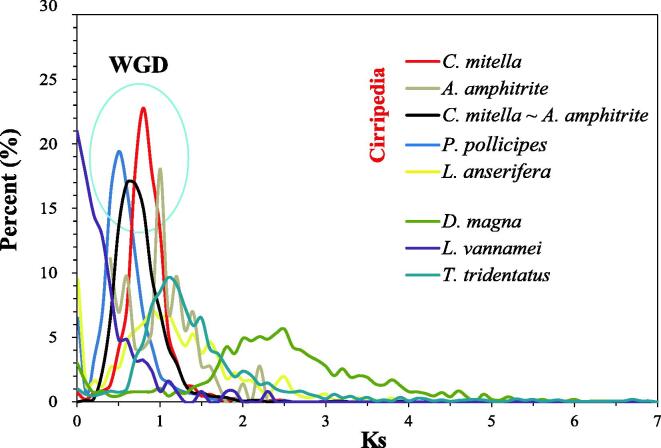
**The distribution of the synonymous substitution rate (Ks) of homologous gene groups for intraspecies and interspecies comparisons.** The peaks in the light blue circle indicate the whole-genome duplication (WGD) events.

The WGD event is likely to be more ancient since similar interchromosomal synteny and duplication patterns in the BUSCOs and Hox gene clusters have also been identified in barnacle genomes other than *C. mitella* (Fig. 2, Fig. 3, Supplementary Fig. S4). To determine the timing of the WGD event, we calculated the distribution of the synonymous substitution rate (Ks) of the paralogous gene groups in the genomes of the four barnacles (Fig. 4). A single Ks peak of the paralogous genes was identified in all four barnacle genomes, which was similar to the WGD genome of *T. tridentatus*. A Ks peak (Ks = 0.6) of orthologous genes between the two barnacle species (*C. mitella* versus *A. amphitrite*) was identified before the Ks peaks of the paralogous genes of *C. mitella* (Ks = 0.8) and *A. amphitrite (*Ks = 1). This result indicates that WGD may have occurred prior to the divergence of the thoracican barnacles.

### Whole-genome duplication facilitates intertidal adaptation in barnacles

Similar to the findings in species with WGD, most of the duplicated genes resulting from the WGD were subsequently lost or underwent non-functionalization in *C. mitella* [41], [42]. Whereas, the remaining duplicated genes provided the potential for functional diversification and adaptive evolution [2], [1]. According to the BUSCO result, more than 55% of the BUSCOs were single-copied in the four barnacle genomes (Supplementary Fig. S4), indicating that at least 55% of the genes had lost their second copies after WGD. The remaining duplicated genes (1675 ohnologs) of the 169 syntenic blocks of *C. mitella* were primarily enriched in many environmental information processing pathways (e.g., adherens junction, cell adhesion molecules, and tight junction) and signal transduction-related pathways (e.g., neuroactive ligand-receptor interaction, axon guidance, olfactory transduction, the neurotrophin signaling pathway, dopaminergic synapse, and many other signaling pathways) (Supplementary Table S8). Many genes involved in these pathways (especially the pathways of neuroactive ligand-receptor interaction and cell adhesion molecules) showed differential expression during the larval settlement and air exposure (Supplementary Table S9) [44]. Thus, these genes are functionally associated with the adaptive evolution of *C. mitella* to the sessile lifestyle and intertidal habitat.

To confirm whether these retained genes play a role in theintertidal adaptation of barnacles, we performed transcriptome sequencing on *C. mitella* under the ebb and flow of the tides. Many genes, including these retained genes, were differentially expressed when exposed to air *vs.* immersed in seawater (Supplementary Fig. S9). Among the 1675 ohnologs of the syntenic blocks of *C. mitella*, 120 genes were not expressed in all samples, and 148 genes displayed significant differential expression (*p* < 0.05), among which 140 genes were differentially expressed in only one of the paired ohnologs. This suggests that approximately 7.16% (120/1675) of the ohnologs underwent nonfunctionalization, and 8.36% (140/1675) of the ohnologs underwent neofunctionalization and/or subfunctionlization. Additionally, an increasing number of genes were up-regulated along with the duration time of air exposure (Supplementary Fig. S9), indicating these retained genes are sensitive to air exposure and should have facilitated the intertidal adaptation of barnacles.

A large number of stress-responsive genes have been reported to play important roles in intertidal adaptation, including cytochrome P450 (CYP450), superoxide dismutase (SOD), heat shock protein (HSP), facilitated trehalose transporter (Tret), glutathione S-transferase (GST), inhibitor of apoptosis protein (IAP), and fibrinogen-related domain-containing protein (FREP) genes [45], [46]. Comparative genomics analysis revealed that genes encoding CYP450, HSP70, and GST had undergone specific expansion and tandem duplication in the barnacle genomes relative to non-intertidal crustaceans (e.g., shrimp and *Daphnia*) (Supplementary Fig. S10-S12), which was similar to that of the intertidal molluscs (e.g., oysters and mussels) (Supplementary Table S10). Thus, the convergent evolution of these stress-responsive genes suggests that they played important roles in the intertidal adaptation of both barnacles and intertidal molluscs. Notably, the genes encoding CYP450 and HSPs were mostly up-regulated upon air exposure (Supplementary Fig. S13, S14). In addition, most of the CYP450 genes (23/65) were involved in the 1675 retained genes after WGD, whereas only two genes encoding HSPs were found among these ohnologs. Therefore, the WGD may have specifically contributed to the duplication of the CYP450 genes and thus facilitated intertidal adaptation in barnacles.

### Subfunctionalization/neofunctionalization after whole-genome duplication

**Fig. 5:**
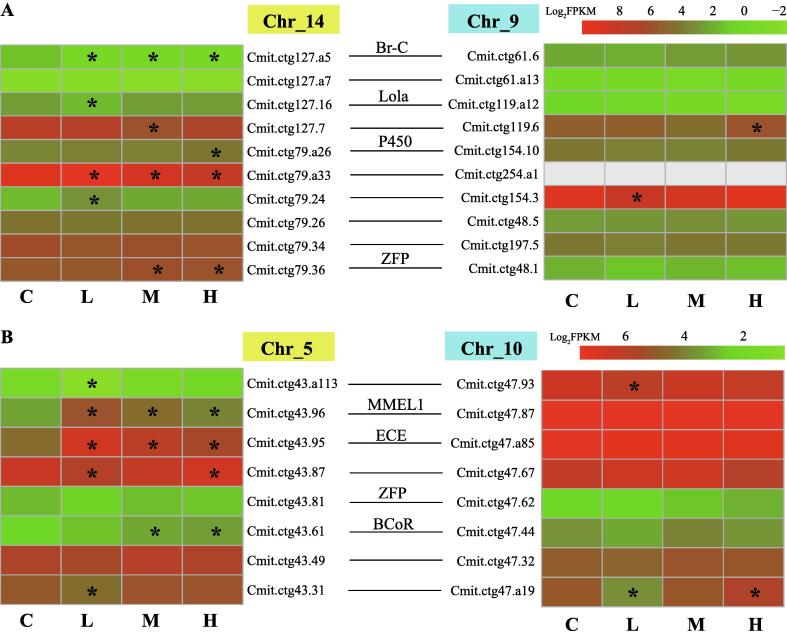
The differential expression patterns of the ohnologs during air exposure in barnacles. (A) The expression patterns of the ohnologs in a syntenic block of two paralogous chromosomes (Chr_14 and Chr_9) that originated from a whole-genome duplication (WGD) event (Fig. 2A). Individuals attached at high (H), medium (M), and low (L) elevation from sea level were sampled, and individuals that were exposed to seawater served as the controls (C). The lines that link two genes indicate that the two genes are ohnologs in the syntenic block. The genes encoding the broad-complex core protein (Br-C), longitudinals lacking protein (Lola), cytochrome P450 (P450), and zinc finger protein (ZFP) are involved in this syntenic block. The other genes are mostly encoding proteins with an unknown function. “*” indicates a significant differential expression in contrast to the control group (student t-test, *p* < 0.05). (B) The expression patterns of the ohnologs in a syntenic block of two paralogous chromosomes (Chr_5 and Chr_10) originated from a WGD event. The genes encoding ZFP, membrane metalloendopeptidase 1 (MMEL1), endothelin-converting enzyme (ECE), and BCL-6 corepressor (BCoR) are involved in this syntenic block.

When analyzing the gene expression patterns of the 1675 ohnologs after WGD in *C. mitella*, it was interesting to find that the paired ohnologs in the syntenic blocks showed differential expression patterns during air exposure. In a syntenic block of two paralogous chromosomes (Chr14 and Chr9) (Fig. 5A), genes encoding CYP450, zinc finger protein (ZFP), broad-complex core protein (Br-C), and longitudinals lacking protein (Lola) were involved and showed differential expression. Interestingly, ohnologs from Chr14 (6 genes) were mostly differentially expressed, whereas ohnologs from Chr9 (2 genes) were less differentially expressed (Fig. 5A). Also, Lola is a broad-complex, tramtrack and zinc finger (BTB-ZF) family transcription factor that is suggested to function in the settlement and environmental signaling processes [25]. In addition, both Lola and Br-C genes have been expanded in the barnacle genomes [25]. These ohnologs with functional similarities were retained in the syntenic blocks of both *C. mitella* and *P. pollicipes* genomes, indicating their conserved and important functions in intertidal adaptation. Genes encoding CYP450, ZFP, Br-C, and Lola were all differentially expressed in the Chr14 block. In contrast, none of their ohnologs showed differential expression in the Chr9 block (Fig. 5A). Similar results were also observed in another syntenic block (Chr5 and Chr10), which also displayed a biased expression pattern between the ohnologs of the two paralogous chromosomes (Fig. 5B). These results indicated that subfunctionalization or neofunctionalization of the ohnologs may have occurred after WGD.

**Fig. 6:**
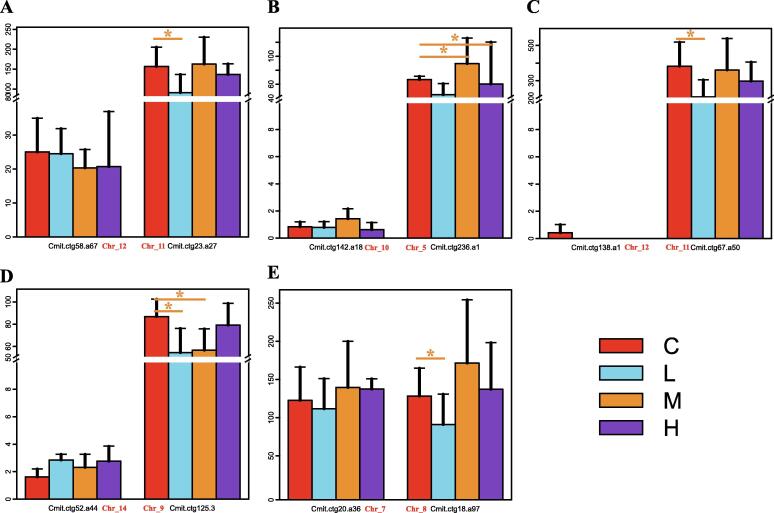
**Genes and metabolites involved in the intertidal adaptation of barnacles.** (A) Volcano plot of differential metabolites between the controls (C) and medium elevation (M) groups of C. mitella . Red and green symbols represent significantly differentially regulated acyl-carnitines and FFAs during air exposure. (B) Schematic diagram of the accumulation of acyl-carnitines in C. mitella during air exposure. Green and red rounded rectangles represent significantly downregulated and upregulated metabolites, respectively. Blue and red rectangles represent the expression levels of the corresponding genes in the control group and the medium group, respectively.

To further test this hypothesis, we focused on the 23 ohnologs encoding CYP450s, because it was the major antistress gene that enriched in the syntenic blocks. The pairwise ohnologs were identified, and their expression patterns were compared. Notably, most of these paired ohnologs showed a significant differential expression pattern, with one having a higher expression level and differential expression upon air exposure, while the other was having a lower expression level and no differential expression (Fig. 6A-D). Only a few paired ohnologs showed similar expression patterns (Fig. 6E). Therefore, a part of CYP450 might have undergone subfunctionalization or neofunctionalization after WGD for intertidal adaptation in barnacles.

### Parallel evolution of genes in relation to the intertidal adaptation

As mentioned above, in addition to CYP450, there were many other stress-responsive genes, including HSPs and GSTs, which also showed a specific expansion in the barnacle genomes compared to the non-intertidal crustacean genomes (Supplementary Table S10, Fig. S10-S12). However, these genes were not present in the 1675 ohnologs of the syntenic blocks; instead, they were mostly distributed as tandem duplicates in the genome. Parallel recruitment of the identical duplicated genes in different species suggests that parallel evolution may have occurred in various barnacles for their intertidal adaptations, since they have similar intertidal inhabitants but different geographical distributions.

HSP70 proteins are the most commonly studied HSPs in intertidal organisms [46], [47]. Among the HSP70 subfamilies, we found that heat shock protein 70B (HSP70B2) was present in both barnacles and intertidal molluscs, but absent in non-intertidal crustaceans (Supplementary Fig. S10). Furthermore, HSP70B2 in *C. mitella* was mostly up-regulated upon air exposure (Supplementary Fig. S13), suggesting its important role in intertidal adaptation. Notably, the HSP70B2 gene underwent intra-species specific expansion after the divergence of three barnacle species (*C. mitella*, *P. pollicipes*, and *A. amphitrite*), as genes from the same species had higher similarity than interspecies, since they clustered together on the phylogenetic tree (Supplementary Fig. S10). Thus, HSP70B2 should have undergone strong parallel evolution in barnacles. In addition to HSPs, genes encoding GSTs were also expanded in barnacles; however, these genes were rarely differentially expressed in *C. mitella* upon air exposure. Similar to HSP70B2, GST Mu also underwent species-specific expansion after the divergence of *C. mitella*, *P. pollicipes*, and *A. amphitrite*. Therefore, parallel evolution had occurred in various barnacle species in terms of intertidal adaptations.

### Metabolomic evidences for the intertidal adaptation in barnacles

**Fig. 7:**
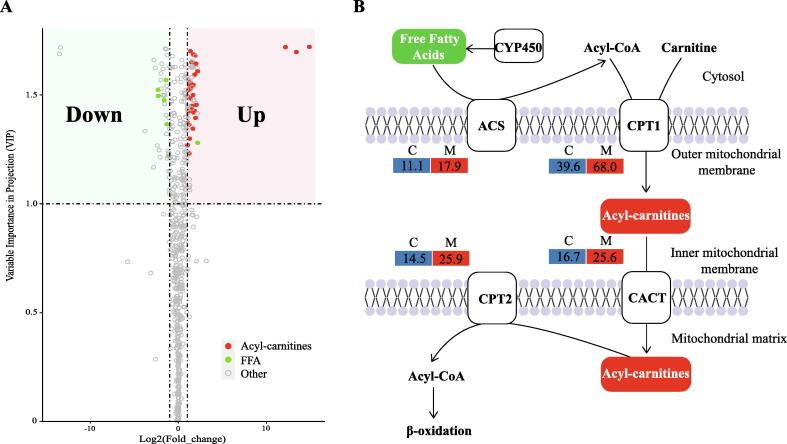

To further understand the mechanism of intertidal adaptation in barnacles, we performed a comparative metabolomic analysis of barnacles under the ebb and flow of tides. The results showed that 71 metabolites were significantly up-regulated upon air exposure, among which acyl-carnitines were the most abundant (34 members, 47.89%) (Fig. 7). Among the 45 down-regulated metabolites, long-chain free fatty acids (FFAs) were the most abundant metabolites (8 members). Acyl-carnitines serve as intermediates of fatty acid conjugation to carnitine (Fig. 7B), and many oxygenation reactions of fatty acids were catalyzed by CYP450s [48], [49], [50]. The accumulation of acyl-carnitines and the down-regulation of FFAs can be explained by the up-regulation of acyl-CoA synthase (ACS) and carnitine palmitoyltransferase 1 (CPT1) (Fig. 7B). Similarly, numerous carnitine-conjugated metabolites (mainly acyl-carnitines) have been shown to accumulate in intertidal molluscs at low tide and under hypoosmotic conditions [51], [52], [53]. Regardless of the accumulated acyl-carnitines used by different species, the similar metabolic features of intertidal barnacles and molluscs indicated that they have a convergent physiological response for intertidal adaptation.

## Discussion

### Signatures of an ancient whole-genome duplication in the last common ancestor of Thoracicalcarea

Despite numerous barnacle genomes have been sequenced in previous studies [22], [23], [24], [25], signatures of WGD have not been examined among them. This study presents robust evidences of a WGD event in barnacles based on the newly assembled genome of *C. mitella*. This is a novel WGD event identified in a single lineage of Crustacea. WGD events have rarely been reported in crustaceans and hexapods, despite the fact that they are the two major groups of arthropods, encompassing the most abundant and diverse species on earth [54]. Among them, the oriental river prawn *M. nipponense* is the only species that has been assumed to have undergone WGD [20]. However, limited syntenic blocks have been identified in the *M. nipponense* genome, and these blocks were randomly distributed among the chromosomes. The present study identified abundant syntenic blocks, an apparent paralogous chromosome relationship, duplicated BUSCO core genes, duplicated Hox gene clusters, and a characteristic Ks peak in the *C. mitella* genome, all of which are consistent with that of the well-known WGD species [41], [42]. In addition, similar results have been identified in other thoracican barnacles, suggesting that this WGD event is more ancient and may have occurred prior to the divergence of Thoracicalcarea (Cirripedia: Thoracica).

Thoracica, Rhizocephala and Acrothoracica are the three major taxa of Cirripedia, with Rhizocephala and Acrothoracica being phylogenetically more ancient than Thoracica [55]. Due to the lack of sequenced genomes of other cirripedes, such as species from Rhizocephala and Acrothoracica, it is impossible to identify WGD events in these species or to infer whether the WGD event predates the divergence of Thoracicalcarea. A previous study indicated that a diploidization event may have occurred during the evolution of a single genus of parasitic barnacles (Cirripedia: Rhizocephala) [21]. Whereas, one species of Acrothoracica (*Trypetesa lampas*) was found to have a diploid chromosome number of 12, and a haploid number of 6 [56], which was less than half the chromosome number of the thoracican barnacles (2n = 32 in general and for *L. anatifera*, 2n = 26) [57]. Chromosome fusions and fissions might have occurred in these cirripedes; thus, whether the chromosomes in Acrothoracica have undergone WGD is unknown. Further genome sequencing of Rhizocephala and Acrothoracica will help clarify this issue.

### Whole-genome duplication and barnacle evolution

Acquisition of evolutionary novelties is a fundamental process of adaptation to the external environment and invasion of new niches, which also leads to life diversification [58]. The WGD event, which creates an additional copy of the entire genome of a species, has shaped the evolutionary history of many lineages, and has been considered as an important source of major evolutionary innovations [59]. During the long evolutionary history, barnacles appear to be one of the most unusual groups of organisms among crustaceans because they have entirely different phenotypes in comparison with other crustaceans, including a calcareous exoskeleton, a sessile lifestyle supported by substrate attachment, and adaptation to an intertidal habitat [60]. The WGD event in barnacles may have contributed significantly to the acquisition of these evolutionary novelties, and driven the origin of barnacles and dramatic changes in their lifestyles.

Our recent study have found that the subfunctionalization and/or neofunctionalization of the expanded duplicate genes contribute to the origin of the calcareous shell and sessile lifestyle [60]. Some of these duplicated genes, such as those encoding alkaline phosphatase (ALP), carbonic anhydrase (CA), settlement-inducing protein complex (SIPC), and cement protein (CP), were identified to have originated prior to the divergence of thoracican barnacles. The divergence of two copies of ALP genes in the ancestral barnacle genomes was supposed to trigger the origin of barnacle shell formation [60]. In the present study, duplicated Hox genes and ohnologs involved in intertidal adaptation were also identified in the ancestral barnacle genome. In both *C. mitella* and *P. pollicipes*, a second copy of *antp* and *ubx* were identified in their genomes. These two genes are located in the middle of the Hox gene cluster, which play important roles in thoracic development in crustaceans [61]. In cirripedes, the thoracic limbs are modified into plumose ‘‘cirri’’, which have specialized functions for the filtration of planktons and respiration [16], [17]. Therefore, barnacles have retained these two duplicated Hox genes (*antp* and *ubx*) after the WGD, which is essential for the evolutionary novelty of their body plan. Taken together, barnacles have evolved numerous novelties in their morphology, physiology, and environmental adaptations, most of which were closely associated with the WGD event in the ancestral barnacles.

The origin and evolution of barnacles is an attractive topic due to their unique morphological and physiological phenotypes. In such a case, Darwin is known to have spent eight years of his life (1846–1854) investigating barnacles [62]. Based on the results of this study, the WGD event was determined to have occurred prior to the divergence of thoracican barnacles, and the duplicated genes caused by the WGD were proposed to construct the unique phenotypes and promote intertidal adaptation in barnacles. According to a previous study, the divergence time of various thoracican barnacles was estimated to be around the Permian-Triassic mass extinction event (∼252 mya) [55], the greatest mass extinction event in the Earth’s history, which resulted in the elimination of more than 90% of marine species [63]. Despite the severity of this extinction, the ecological recovery of marine invertebrates following the event was explosive and occurred over a relatively short period during the Early Triassic [63]. The dramatic change in eustatic sea levels is one of the major causes for the extinction and recovery events, which opportunistically provided sufficient new niches for intertidal organisms, including thoracican barnacles [64], [65]. Thus, both the harsh marine conditions during the mass extinction and the refined conditions after the extinction may have contributed to the origin, diversification and evolution of thoracican barnacles. The WGD has been regarded as an advantageous event in changing the physiology and morphology of species, leading to an adaptation to the surrounding environment and evolutionary innovations [58]. In barnacles, the duplicated genes related to calcareous shell formation, settlement, and intertidal adaptation seem to contribute to the innovations of physiology, morphology, and intertidal adaptation. In such cases, the occurrence of the WGD event in the ancestor of barnacles is likely to be a consequence of barnacle evolution and a potential driving force for the origin and diversification of thoracican barnacles.

### Subfunctionalization/neofunctionalization after whole-genome duplication promotes intertidal adaptation

Nonfunctionalization of genes is the most likely outcome of WGD, which is caused by the accumulation of deleterious mutations in coding regions of the genes due to the lack of selective pressure to maintain their functions [58]. Although most duplicated genes resulting from WGD have undergone nonfunctionalization, the remaining duplicated genes provide the potential for subfunctionalization or neofunctionalization, and thus contributing to functional diversification [2], [1]. As expected, the duplicated genes retained in *C. mitella* were primarily enriched in many environmental information processing pathways, which should contribute to the adaptive evolution of *C. mitella* to a sessile lifestyle and intertidal habit. Both subfunctionalization and neofunctionalization can occur because of the relaxation of selective pressures, and duplicated genes in both processes may split functions and diverge to generate a new function, respectively [66]. Our study provides evidences for the possible subfunctionalization and neofunctionalization among ohnologs during their intertidal adaptation.

Since barnacles and some molluscs (e.g., oysters and mussels) are sessile organisms living in the intertidal zone, they should be subjected to similar selective pressures and adopt similar mechanisms for their intertidal adaptation. In this study, some antistress factors, including CYP450s, HSPs, and GSTs, were found to have undergone specific expansion in barnacles and intertidal molluscs relative to their non-intertidal relatives. As indicated by previous studies, the functions of CYP450s have been certified in oysters, where they are important molecules for the biotransformation of endobiotic and xenobiotic chemicals, thus contributing to their adaptation to the intertidal environment [45], [67]. Coincidentally, FFAs are one of the major endobiotic targets for CYP450 biotransformation [50], and FFA and its intermediates (acyl-carnitine) were also the major differentially regulated metabolites during barnacle intertidal acclimation (Fig. 7). In addition, acyl-carnitines of intertidal molluscs have been shown to have a rhythmic accumulation profile that peaked during air exposure at low tide, which suggested that the accumulation of acyl-carnitines may be a consequence or mechanism of intertidal adaptation [51], [52], [68]. HSP70s have been identified to be important molecular chaperones for barnacles adapting the thermal intertidal zone [69]. The co-expansion of these antistress factors in barnacles and intertidal molluscs suggests the presence of strong convergent evolution for intertidal adaptation.

However, there are some differences between barnacles and intertidal molluscs. Barnacles have undergone a WGD event for adaptive evolution to their intertidal sessile lifestyle, whereas WGD events have seldom been reported in Mollusca [70]. When we examined the signatures of WGD in these expanded antistress genes, it was interesting to find that not all of these genes (e.g., HSPs and GSTs) oringinated from WGD. Most of the genes encoding the HSPs and GSTs were tandemly duplicated in the genomes, which is a different duplication event distinct from WGD [71]. CYP450s are one of the largest families of proteins that control various physiological processes via biosynthetic and detoxification pathways, and some CYP450s are known to play a central role in the adaptation of animals to stressful environments [72]. The genes encoding CYP450s were the major gene family that expanded after the WGD event in barnacles. Many, but not all, CYP450 genes showed a specific response to the stress of air exposure. The divergence of their expression patterns suggested that subfunctionalization or neofunctionalization have happened in these ohnologs.

Subfunctionalization refers to the process by which two members of ohnologs each assumes a subset of the functions previously carried out by the ancestral singleton gene. While neofunctionalization describes the diversification of one member of ohnologs, enabling it to acquire a new function [66]. Limited knowledge is available about the specific functions of these differentially expressed CYP450 ohnologs, making it difficult to define what constitutes a “new” function for a member of the duplicated gene pairs [73]. Nevertheless, these functional differentiations may somehow confer an advantage to the organism, resulting in the ancient duplicated pair of genes becoming fixed in the lineage by natural selection.

Functional divergence of gene pairs after WGD has been reported in many species, which ultimately contributes to phenotypic innovation and organismal diversification [2], [5], [74], [75]. In comparison with other crustaceans, the lifestyle of barnacles changed significantly from free-swimming to permanent settlement in the intertidal zone. Consequently, subfunctionalization or neofunctionalization of CYP450 genes and other antistress genes may have played an important role in the lifestyle transition of barnacles. Therefore, further studies on the functional divergence of these genes will help us understand the origin of the sessile lifestyle and the key molecular mechanisms underlying intertidal adaptation in barnacles.

### Genetic changes underlying the parallel adaptation to the intertidal zone of various barnacles

Parallelism is a form of independent evolution of similar or identical traits, which is often taken as evidence for adaptive evolution [76]. Unlike duplicated genes of WGD, which are derived from the ancestral genome, some genes are duplicated in parallel in geographically separated groups of organisms [77]. In barnacles, genes encoding HSP70B2 and GST Mu have been identified to undergo parallel evolution after the divergence of various barnacles since these barnacles are geographically distinct. Similar to CYP450s, these genes also play an important role in intertidal adaptation. Whereas, unlike CYP450s, which with divergent expression patterns in ohnologs, the genes encoding HSP70B2 and GST Mu all showed similar expression patterns. Thus, these genes are responsible for similar but independently evolved adaptations. Beside intertidal adaptation, parallel evolution has also been identified in genes related to shell formation and settlement in barnacles [60]. A key biomineralization gene, *ALP*, has undergone parallel duplications in both stalked and sessile barnacles, which is supposed to be crucial for the origin and adaptive evolution of the calcareous exoskeleton in barnacles. Similar results had been found in the *CP*s for sessile life adaptation [2], [60]. Therefore, in addition to WGD events, parallel evolution should also play an important role in the adaptive evolution of barnacles.

## Conclusion

In conclusion, the present study provided strong evidence of a WGD event in barnacles based on our newly assembled genome of *C. mitella*. This is a novel WGD event that has been identified in a single lineage of Crustacea. This WGD event occurred in the ancestor of barnacles, which has significant implications for understanding the genome evolution of Cirripedia. Subfunctionalization and/or neofunctionalization were identified in paralogous chromosomes following the WGD, potentially driving the diversification of barnacles. The duplicated CYP450 ohnologs that originated from the WGD contribute to the adaptive evolution of their intertidal sessile lifestyle. The findings of the WGD in this study shed light on the origin and diversification of barnacles and their adaptive evolution of unique phenotypes.

## Compliance with ethics requirements

The methods were performed in accordance with relevant guidelines and regulations and approved by the Animal Ethics Committee [2020(37)] at Institute of Oceanology, Chinese Academy of Sciences.

## Declaration of Competing Interest

The authors declare that they have no known competing financial interests or personal relationships that could have appeared to influence the work reported in this paper.
