# Supplementary material for: An ancient whole-genome duplication in barnacles contributes to their diversification and intertidal sessile life adaptation
Source: J Adv Res. 2023 Sep 20;62:91–103. doi: 10.1016/j.jare.2023.09.015 (PMC11331182; doi:10.1016/j.jare.2023.09.015)
Supplement: Supplementary data 1 [file mmc1.pdf]

Supplementary materials for

An ancient whole-genome duplication in barnacles contributes to their diversification and intertidal sessile life adaptation

Jianbo Yuan[1,2,#], Xiaojun Zhang[1,2,#], Xiaoxi Zhang[1,#], Yamin Sun[3,#], Chengzhang Liu[1,2], Shihao Li[1,2], Yang Yu[1,2], Chengsong Zhang[1,2], Songjun Jin[1,2], Min Wang[4,5,*], Jianhai Xiang[1,2,*], Fuhua Li[1,2,*]

[1] CAS and Shandong Province Key Laboratory of Experimental Marine Biology, Center for Ocean Mega-Science, Institute of Oceanology, Chinese Academy of Sciences, Qingdao 266071, China.

[2] Laboratory for Marine Biology and Biotechnology, Qingdao National Laboratory for Marine Science and Technology, Qingdao 266237, China.

[3] Research Center for Functional Genomics and Biochip, Tianjin 300457, China.

[4] TEDA Institute of Biological Sciences and Biotechnology, Nankai University, Tianjin 300457, China.

[5] Key Laboratory of Molecular Microbiology and Technology, Ministry of Education, Nankai University, Tianjin 300071, China.

[#]These authors contributed equally. [*]Correspondence and requests for materials should be addressed to F.L. (fhli@qdio.ac.cn, Orcid ID: 0000-0001-8693-600X), or to J.X. (jhxiang@qdio.ac.cn, Orcid ID: 0000-0001-5395-7787), or to M.W. (wangm@nankai.edu.cn).

Supplementary Notes:

Supplementary Note 1. Genome sequencing and assembly

The genomic DNA was sheared using a sonication device for the construction of short-insert (500 bp) paired-end (PE) libraries. All libraries were sequenced by using the HiSeq X-TEN sequencing platform (Illumina, San Diego, CA, USA). The Illumina sequence adaptors and the low-quality reads were trimmed using Trimmomatic (v.0.35). For PacBio library construction, genomic DNA of the same individual was sheared into fragments with a length of ~20kb, and fragments shorter than 7 kb were filtered out using BluePippin (Sage Science, MA, USA). The filtered DNA was then converted into a SMRTbell library using the PacBio DNA Template Preparation Kit following the manufacturer's instructions. Single Molecule Real Time (SMRT) sequencing was conducted on a PacBio Sequel sequencing platform (Pacific Biosciences, Menlo Park, CA, USA) using the V3.0 sequencing reagent and a SMRT Cell (1 M, V3) (Supplementary Table S1).

To assemble the *C. mitella* genome into chromosome-level, a Hi-C library was constructed to anchor the contigs. The sample of another individual was flash frozen and pulverized prior to formaldehyde crosslinking, and then digested by restriction enzyme (*Mbo* I/*Dpn* I/*Hin*d III). The ends were labeled with biotin and then supplemented and connected. Cross-links were removed by proteinase K and SDS, and DNA was extracted by AMPure XP beads. The labeled DNA fragments were captured with M-280 streptavidin beads, and the ends were repaired by adding A-tails. Finally, the constructed library was used for sequencing on the HiSeq X-TEN platform (Illumina, San Diego, CA, USA).

The *C. mitella* genome was *de novo* assembled using WTDBG2 with the default parameters based on the subreads from PacBio sequencing [1]. The assembled contigs were then polished by Quiver (SMRT Analysis v2.3.0). Besides, we performed several rounds of iterative error corrections using the clean data from Illumina. To assemble the chromosome-level genome, the Hi-C sequencing data were aligned to the assembled contigs using Juicer [2]. The 3D-DNA pipeline (version 180419) was used to assign the order and orientation of each group [3], and the contact heatmaps

were plotted using HiCPlotter [4].

To evaluate the quality of the genome assembly, the Illumina PE reads were mapped back to the assembly using Bowtie2 with the following parameters "--rdg 3,1 --rfg 3,1 --gbar 2" [5]. To evaluate the completeness of the assembly, we mapped the unigenes from the transcriptome data to the *C. mitella* genome (transcriptome sequencing and assembly are described below). In addition, the quality of the genome assembly was also evaluated by benchmarking universal single-copy ortholog (BUSCO) analysis (http://gitlab.com/ezlab/busco). The 1066 conserved BUSCOs of Arthropoda were used as the database for the BLAST search.

Based on the genome sequencing data in the form of 49 Gb of Illumina paired-end reads (96×) and 57 Gb of PacBio long reads (112×, Supplementary Table S1), a high-quality genome of the stalked barnacle *C. mitella* was assembled. The assembled genome size was 512.06 Mb (Supplementary Table S2), which was close to the size estimated by K-mer analysis (513.60 Mb, Supplementary Fig. S2). The assembly showed high continuity, with a contig N50 length of 3.22 Mb, which is among the longest for a crustacean assembly (Supplementary Table S3). The contigs of *C. mitella* were further anchored to 16 chromosomes using high-throughput chromosome conformation capture (Hi-C) sequencing data (277×, Supplementary Fig. S3). More than 93% of the contigs were anchored and the N50 length of the final chromosome-level genome assembly was 30.73 Mb (Supplementary Table S2). The assembly showed high integrity and quality, based on benchmarking universal single-copy ortholog (BUSCO) analysis (94.74%) and unigenes from transcriptomes (91.82%) (Supplementary Fig. S4, Tables S4, S5).

Supplementary Note 2. Genome annotation

For transposable element (TE) annotation, both RepeatModeler and RepeatMasker were used for *de novo* identification and classification [6]. To ensure the integrity of the protein-coding genes in subsequent gene prediction and annotation, we masked all repeat sequences (except low complexity and simple repeats) from this analysis.

For protein-coding gene annotation, a combination of homology-based prediction, *ab initio* prediction and transcriptome-based prediction methods was utilized. *Ab initio* prediction was conducted using Augustus v2.5.5 [7]. For homology-based prediction, we downloaded homologous protein sequences in *A. amphitrite*, *Acyrthosiphon pisum*, *Anopheles gambiae*, *Bombyx mori*, *Daphnia pulex*, *Drosophila melanogaster*, *Eurytemora affinis*, *Eulimnadia texana*, *Ixodes scapularis*, *Litopenaeus vannamei*, *Locust migratoria*, *Parhyale hawaiensis*, *Procambarus virginalis*, *Strigamia maritima*, *Tigriopus californicus* and *Tetranychus urticae*, from the NCBI database, and aligned them to the assembled genome with Exonerate v2.2.0 (http://www.ebi.ac.uk/~guy/exonerate/). For transcriptome-based prediction, the RNA-Seq data were mapped against the assembly using HISAT2 v2.1.0 [8], and then, the transcripts were converted to gene models using Cufflinks v2.2.1 [9]. Finally, we integrated the prediction results from the above three methods using EVidenceModeler (EVM) and obtained a nonredundant gene set [10].

Functional annotations of the predicted genes were conducted using BLASTP against the NCBI-NR, SwissProt, and KOG databases with an E-value of $1E^{-05}$. Protein domains were mapped against the InterPro and Pfam databases by InterProScan and HMMER [11,12]. KEGG Automatic Annotation Server (KAAS) was used to annotate the pathways potentially including the genes by mapping against the KEGG database. The Gene Ontology (GO) terms for each gene were extracted from the corresponding InterProScan or Pfam results. Functional enrichment analysis was conducted on a subset of genes according to their GO and KEGG classifications. The enriched GO terms and KO pathways were calculated relative to the background of all protein-coding genes using OmicShare Cloud Tools (http://www.omicshare.com/).

Repeat annotation revealed that 33.44% of the *C. mitella* genome was composed of repetitive sequences, which was a lower percentage than that found in the genome of the acorn barnacle *A. amphitrite* (43.83%, Supplementary Table S6. *C. mitella* harbored more DNA transposons (5.91%), whereas *A. amphitrite* harbored more simple sequence repeats (3.54%). Among the identified DNA transposons, hAT (2.16%) and TcMar (1.04%) were the two most common transposon types, and most

of these transposons expanded in a relative recent time (Supplementary Fig. S5, S6).

Genome annotation combined gene evidence from homology-based prediction, *ab initio* prediction and transcriptome-based prediction methods. We predicted 13,364 protein-coding genes in the *C. mitella* genome, with an average length of 1,554 bp and an average of 7.32 exons per gene (Supplementary Table S7). BUSCO analysis indicated a high completeness (93.15%) of the annotated genes in *C. mitella*, which was comparable to or better than the completeness of many other reported crustacean genomes[13].

Supplementary Note 3. Phylogenetic analysis

We next performed phylogenetic analysis and divergence time estimation of C. mitella with 10 other arthropods to confirm its placement and divergence time within this family (Supplementary Fig. S3). A total of 127 conserved single-copy orthologous genes among 11 arthropod species were used for phylogenetic tree construction. For each ortholog group, the amino acid sequences were aligned using MUSCLE v3.8.31 with the default settings [14]. All the alignment results were merged to form a super alignment matrix. Then, The maximum likelihood (ML) method was used for the phylogenetic analysis in RAxML with the default parameters [15]. The maximum likelihood phylogeny and respective branch lengths were inferred with RAxML using 1,000 bootstrap replicates. The molecular clock and divergence times were estimated using a combined analysis with the programs of r8s and RAxML [16]. The fossil-derived timescale and the evolutionary history of these species were obtained from TIMETREE [17].

Based on the 127 conserved single-copy orthologous genes among 111 arthropod species, a high consensus phylogenetic tree was constructed. The two barnacle species (*C. mitella* and *A. amphitrite*) was paraphyletic with malacostraceans and nested by other crustaceans (Supplementary Fig. S3). Then, we estimated the molecular clock and the divergence times using a combined analysis of the programs of r8s and RAxML[16]. *C. mitella* is a stalked barnacle in the suborder Lepadomorpha, which is estimated to have diverged from the other major group of thoracican barnacles (sessile

barnacles, Sessilia, represented by *A. amphitrite*) approximately 237 million years ago (mya), just after the Permian-Triassic mass extinction event (~ 252 mya). The thoracican barnacles are estimated to have diverged from other crustaceans approximately 521 mya (Supplementary Fig. S3), close to the timing of the origin of molluscs (the Early Cambrian)[18]. In addition, molluscs started to exhibit mineralization at the dawn of the Cambrian Period[19]. This is consistent with the hypothesis that the biological mechanisms of biomineralization may have evolved at nearly the same time, as biomineralization is the most visible aspect of the so-called "Cambrian explosion"[20]. Therefore, the calcareous shells of barnacles and molluscs may have originated at the same time during the Cambrian.

Supplementary Note 4. Gene family expansion and contraction of the *C. mitella* genome

Gene family clustering was performed on the protein-coding genes of *C. mitella* and 10 additional arthropods, and the gene gain and loss events were identified along each branch of the phylogenetic tree. A large number of Cirripedia expanded gene families (1,131 gene families) were identified. Functional enrichment analysis indicated that the expanded gene families of the four barnacle genomes were primarily enriched in KEGG terms of cytosome, MAPK, and Hedgehog signaling pathways, many pathways related to nervous transduction (e.g., neuroactive ligand-receptor interaction, glutamatergic synapse and serotonergic synapse), inflammatory mediator regulation of transient receptor potential (TRP) channels, Cell adhesion molecules (CAMs), tight junction, and so on (Supplementary Fig. S7). Neuroactive ligand-receptor interaction and neurotransmitters (e,g, serotonin and glutamate) have been identified to play important roles in larval settlement and environment adaptation [21-23]. Cell adhesion molecules (CAMs) and proteins involved in adherens junctions and tight junctions might be very important for their unique sessile lifestyle. Glutamatergic synapse and serotonergic synapse is important in the normal activity of the nervous system[24]. TRP channels exhibit a unique response to temperature that mediates thermal tolerance and have been identified as diagnostic biomarkers of thermal stress in oysters[25]. Therefore, the expansion of these gene

families may reflect the adaptive evolution of barnacles related to the shell formation, sessile lifestyle and inhabiting stressful intertidal zones.

References:

1. Ruan, J. & Li, H. Fast and accurate long-read assembly with wtdbg2. *Nat Methods* **17**, 155-158 (2020).
2. Durand, N.C. *et al.* Juicer Provides a One-Click System for Analyzing Loop-Resolution Hi-C Experiments. *Cell Systems* **3**, 95-98 (2016).
3. Dudchenko, O. *et al.* De novo assembly of the Aedes aegypti genome using Hi-C yields chromosome-length scaffolds. *Science* **356**, 92-95 (2017).
4. Akdemir, K.C. & Chin, L. HiCPlotter integrates genomic data with interaction matrices. *Genome Biol* **16**, 198 (2015).
5. Langmead, B. & Salzberg, S.L. Fast gapped-read alignment with Bowtie 2. *Nature Methods* **9**, 357-9 (2012).
6. Tarailo-Graovac, M. & Chen, N. Using RepeatMasker to identify repetitive elements in genomic sequences. *Curr Protoc Bioinformatics* **Chapter 4**, Unit 4 10 (2009).
7. Stanke, M., Steinkamp, R., Waack, S. & Morgenstern, B. AUGUSTUS: a web server for gene finding in eukaryotes. *Nucleic Acids Research* **32**, W309-12 (2004).
8. Pertea, M., Kim, D., Pertea, G.M., Leek, J.T. & Salzberg, S.L. Transcript-level expression analysis of RNA-seq experiments with HISAT, StringTie and Ballgown. *Nature Protocols* **11**, 1650-1667 (2016).
9. Trapnell, C., Pachter, L. & Lsalzberg, S. TopHat: discovering splice junctions with RNA-Seq. *Bioinformatics* **25**, 1105 (2009).
10. Haas, B. *et al.* Automated eukaryotic gene structure annotation using EVidenceModeler and the Program to Assemble Spliced Alignments. *Genome biology* **9**, R7 (2008).
11. Zdobnov, E.M. & Apweiler, R. InterProScan--an integration platform for the signature-recognition methods in InterPro. *Bioinformatics* **17**, 847-8 (2001).
12. Prakash, A., Jeffryes, M., Bateman, A. & Finn, R.D. The HMMER Web Server for Protein Sequence Similarity Search. *Curr Protoc Bioinformatics* **60**, 3 15 1-3 15 23 (2017).
13. Yuan, J.B. *et al.* Simple sequence repeats drive genome plasticity and promote adaptive evolution in penaeid shrimp. *Communications Biology* **4**(2021).
14. Edgar, R.C. MUSCLE: multiple sequence alignment with high accuracy and high throughput. *Nucleic Acids Res* **32**, 1792-7 (2004).
15. Stamatakis, A. RAxML version 8: a tool for phylogenetic analysis and post-analysis of large phylogenies. *Bioinformatics* **30**, 1312-3 (2014).
16. Sanderson, M.J. r8s: inferring absolute rates of molecular evolution and divergence times in the absence of a molecular clock. *Bioinformatics* **19**, 301-302 (2003).

17. Kumar, S., Stecher, G., Suleski, M. & Hedges, S.B. TimeTree: A Resource for Timelines, Timetrees, and Divergence Times. *Molecular Biology and Evolution* **34**, 1812-1819 (2017).
18. Vinther, J. The origins of molluscs. *Palaeontology* **58**, 19-34 (2014).
19. Marin, F., Luquet, G., Marie, B. & Medakovic, D. Molluscan shell proteins: Primary structure, origin, and evolution. *Current Topics in Developmental Biology, Vol 80* **80**, 209-276 (2008).
20. Peters, S.E. & Gaines, R.R. Formation of the 'Great Unconformity' as a trigger for the Cambrian explosion. *Nature* **484**, 363-366 (2012).
21. Yan, G. *et al.* Comparative Transcriptomic Analysis Reveals Candidate Genes and Pathways Involved in Larval Settlement of the Barnacle Megabalanus volcano. *Int J Mol Sci* **18**(2017).
22. Chen, Z.F. *et al.* Quantitative proteomics study of larval settlement in the Barnacle Balanus amphitrite. *PLoS One* **9**, e88744 (2014).
23. Sun, X.J. *et al.* Transcriptome and metabolome analyses provide insights into the salinity adaptation of clam Ruditapes philippinarum. *Aquaculture Reports* **27**(2022).
24. Pacei, F. *et al.* The Relevance of Thiamine Evaluation in a Practical Setting. *Nutrients* **12**(2020).
25. Fu, H. *et al.* Transient Receptor Potential (TRP) Channels in the Pacific Oyster (Crassostrea gigas): Genome-Wide Identification and Expression Profiling after Heat Stress between C. gigas and C. angulata. *Int J Mol Sci* **22**(2021).

Supplementary Figures

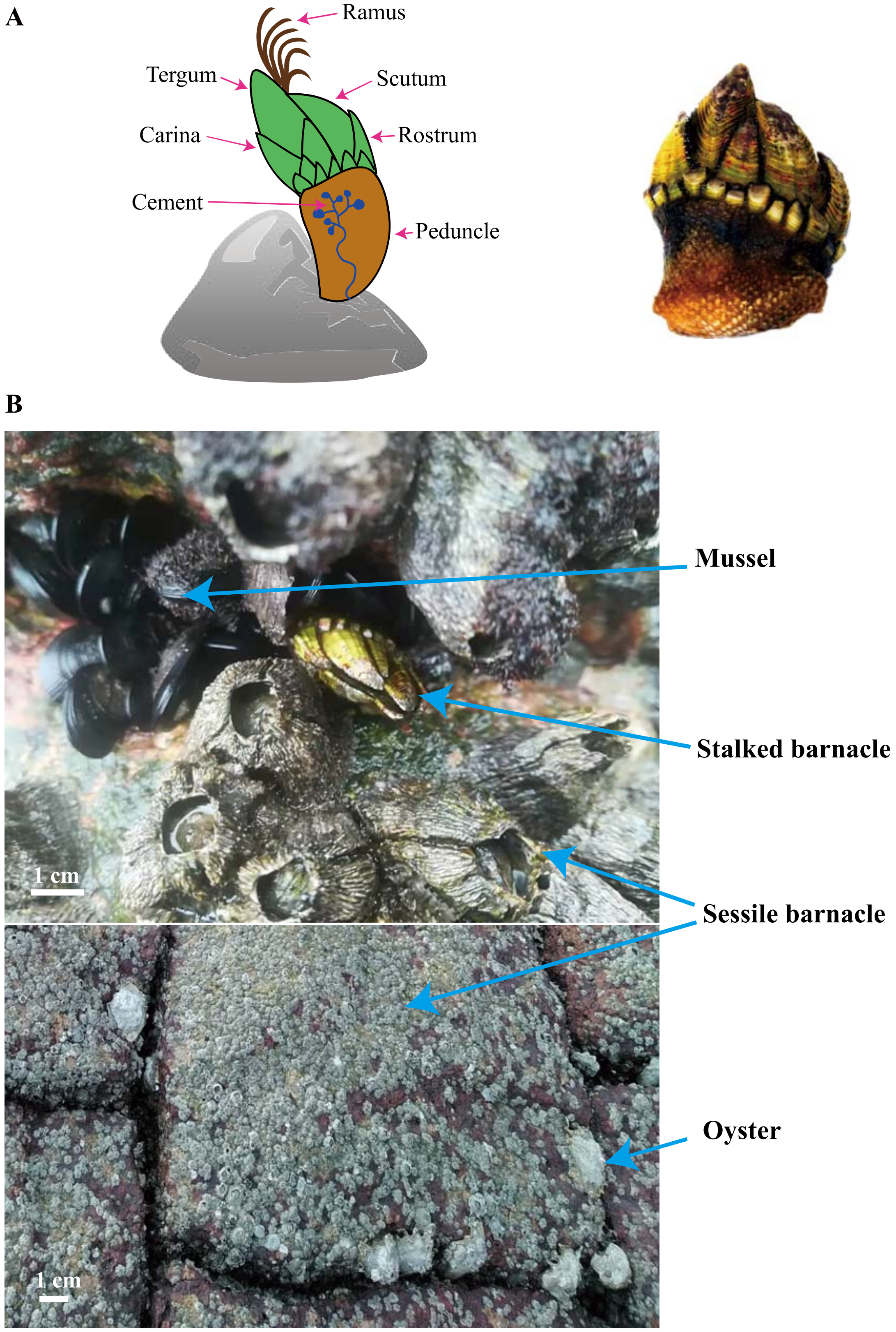

Figure S1. Morphology of the stalked barnacle *C. mitella*. (A) Morphology of *C. mitella*. **(B)** Environment inhabited by *C. mitella*. Stalked barnacles, sessile barnacles and oysters coexist in the same environment.

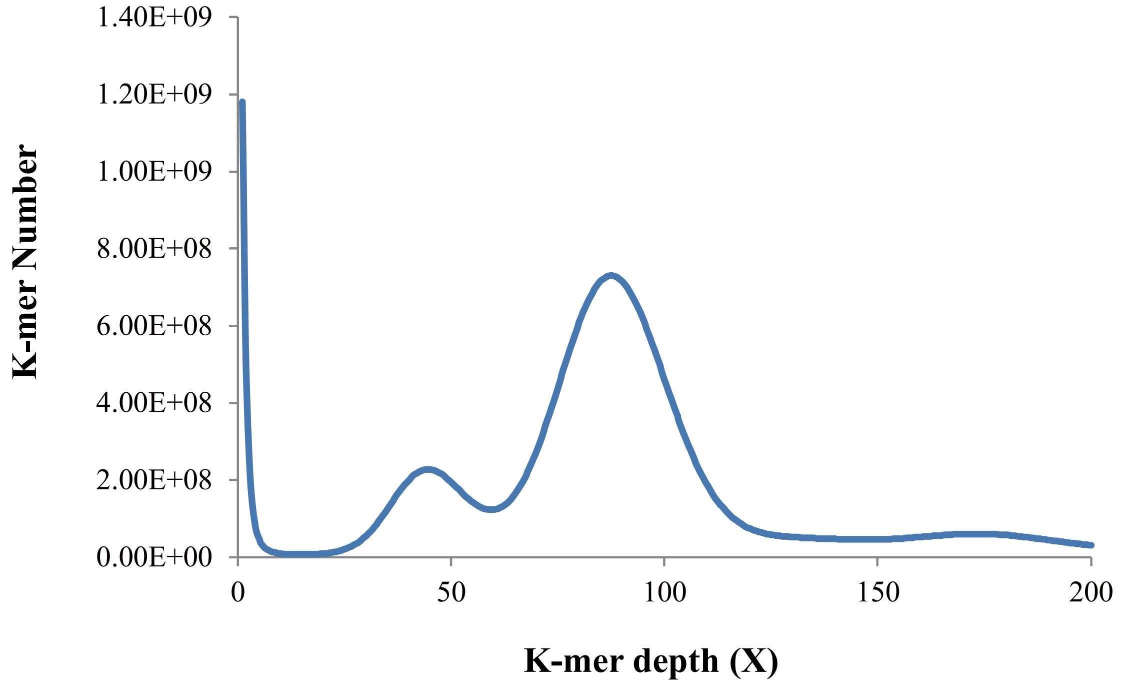

Figure S2. K-mer distribution of the Illumina sequencing data. K-mer analysis estimated the genome size of *C. mitella* to be 513.60 Mb, and repeat content to be 28.76%.

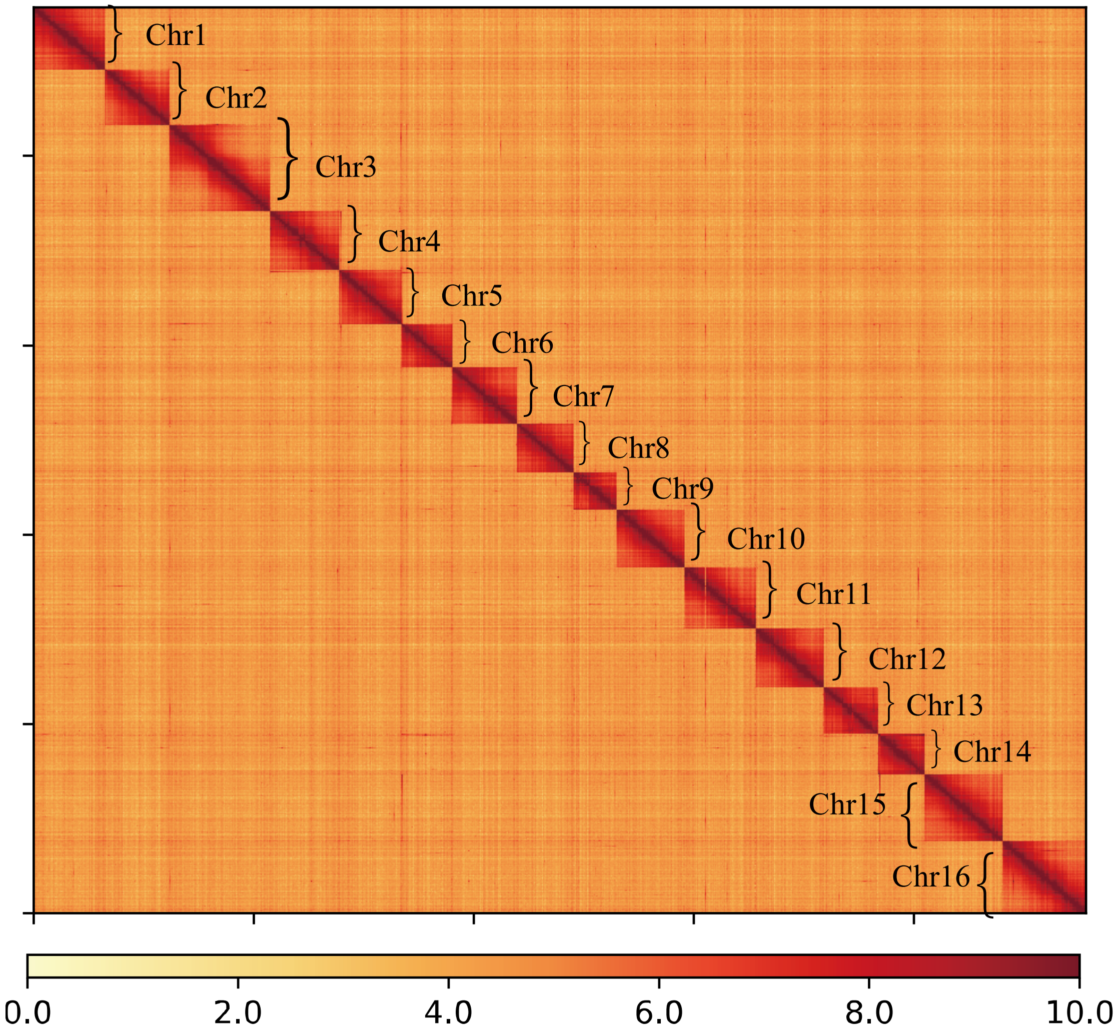

Figure S3. Heatmap of the Hi-C assembly. The color bar indicates the contact density, ranging from white (low) to red (high).

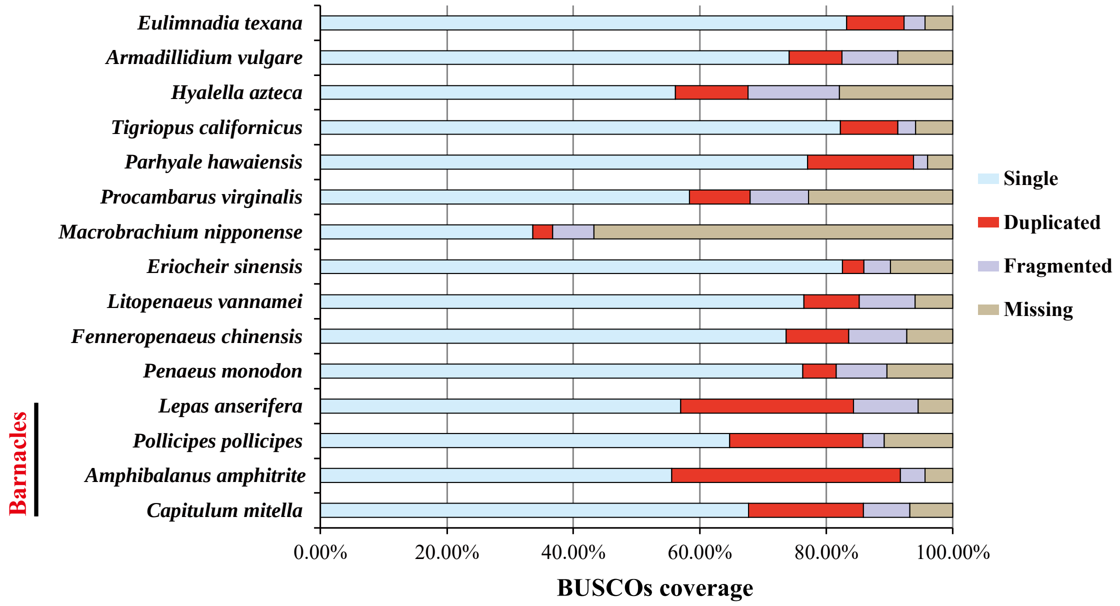

Figure S4. Core gene coverage of the genomes of barnacles and other crustacean relatives. The database used for BUSCO assessment was composed of 1066 BUSCOs of Arthropoda.

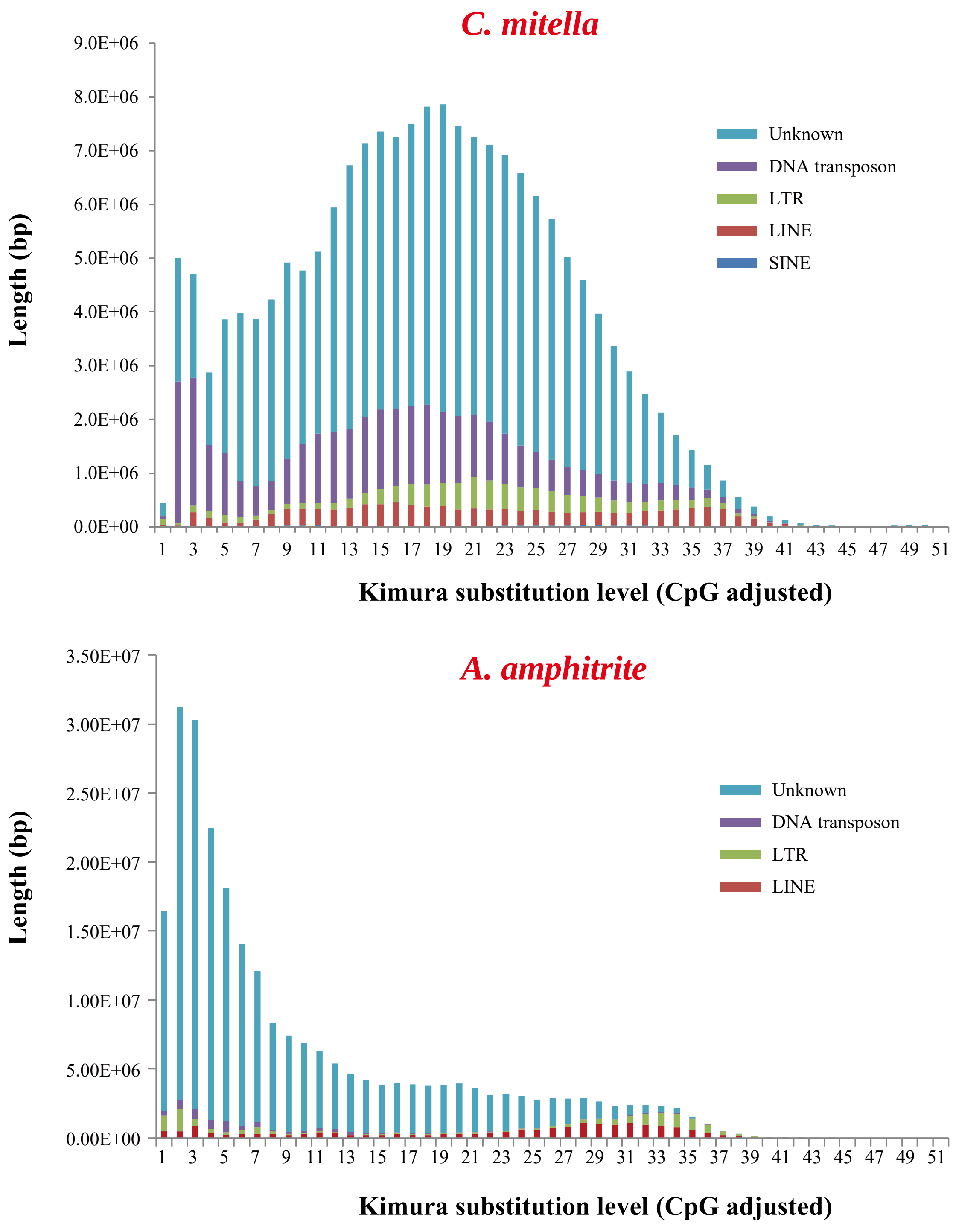

Figure S5. Substitution rate distribution of repeats in the *C. mitella* and *A. amphitrite* genomes.

The substitution rates were calculated between the genomic and repeat consensus sequences using RepeatMasker.

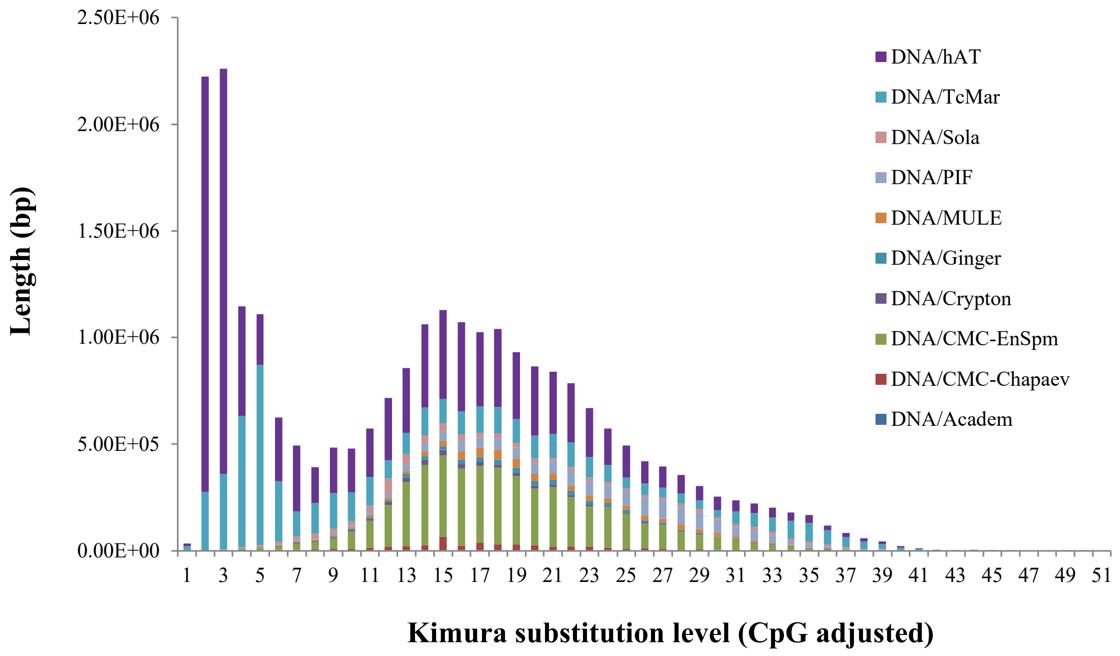

Figure S6. Substitution rate distribution of DNA transposons in the *C. mitella* genome.

The substitution rates were calculated between the genomic and repeat consensus sequences using RepeatMasker. hAT, TcMar and CMC-EnSpm were the three most common transposons in the *C. mitella* genome. hAT and TcMar expanded recently, and CMC-EnSpm showed ancient expansion.

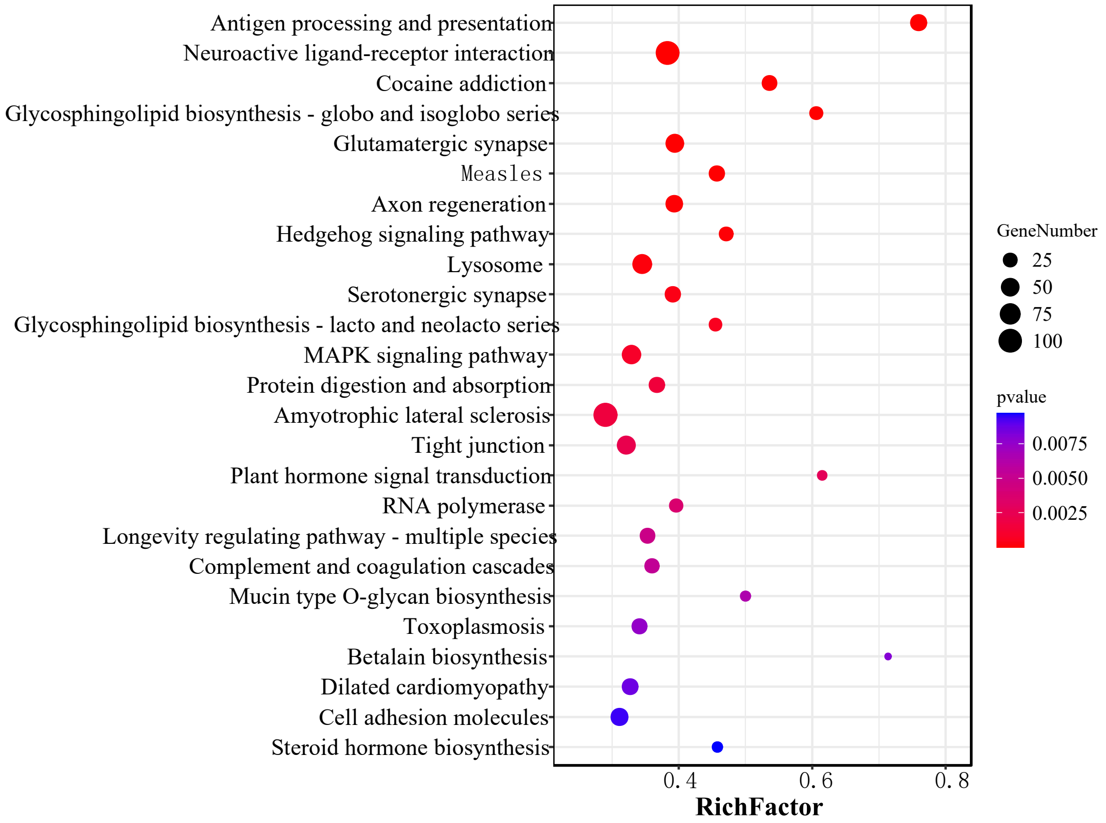

Figure S7. Kyoto Encyclopedia of Genes and Genomes (KEGG) enrichment analysis of the expanded gene families in the fourbarnacle genomes.

The expansion of the gene families was calculated using CAFÉ by comparing differences in cluster sizes between each of the current species and their respective ancestors. The top 25 KEGG pathways under significant enrichment of the expanded gene families are shown in the plot.

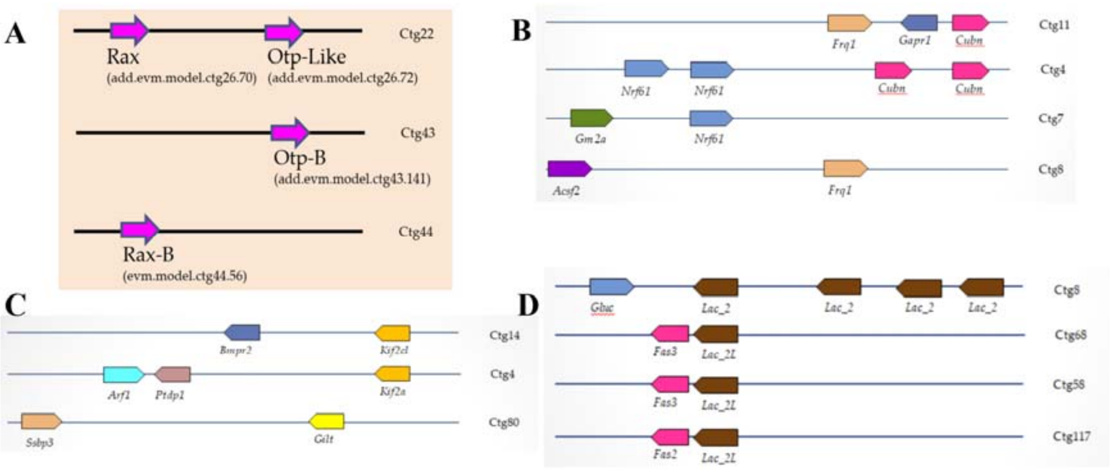

Figure S8. Representative conserved gene clusters with second or multiple copies in the *C. mitella* genome.

(A) The conserved gene cluster "Rax-Hbn-Otp" is incomplete. Only "Rax-Otp" exists on Ctg22, and the other two Rax and Otp gene copies exist on different contigs (Ctg43 and Ctg44). (B) Conserved paralogous "*NotchL* loci". (C) Conserved paralogous "*Pde4L* loci". (D) Conserved paralogous "*Fas1L* loci".

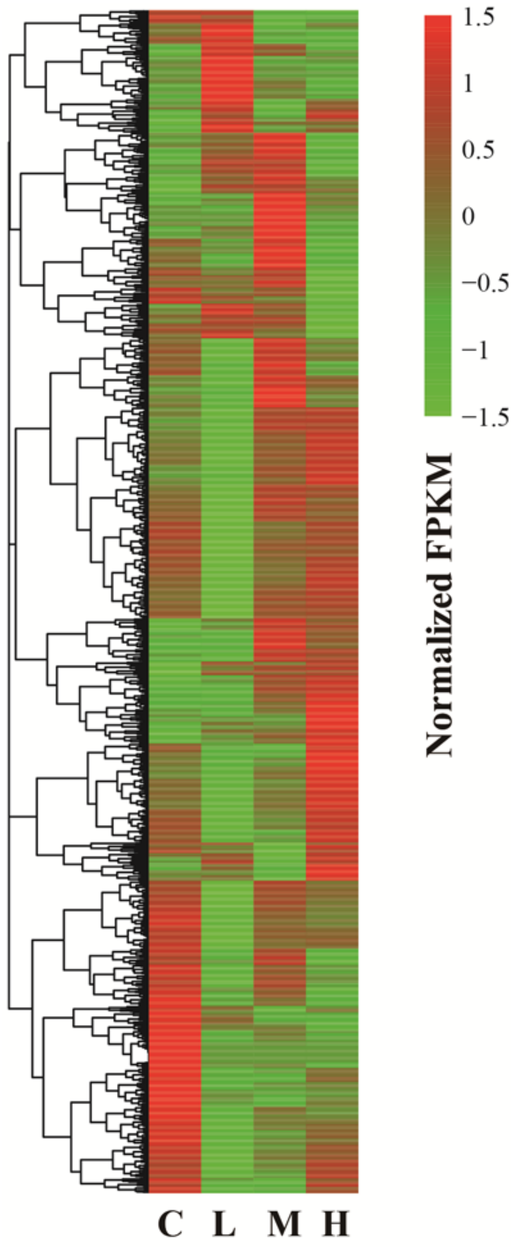

Figure S9. The expression patterns of ohnologs in *C. mitella* during air exposure. C, L, M and H at the bottom represent the samples collected underwater (control, C) and at low (L), middle (M) and high (H) sites from the water level, respectively. The expression levels are normalized for each gene.

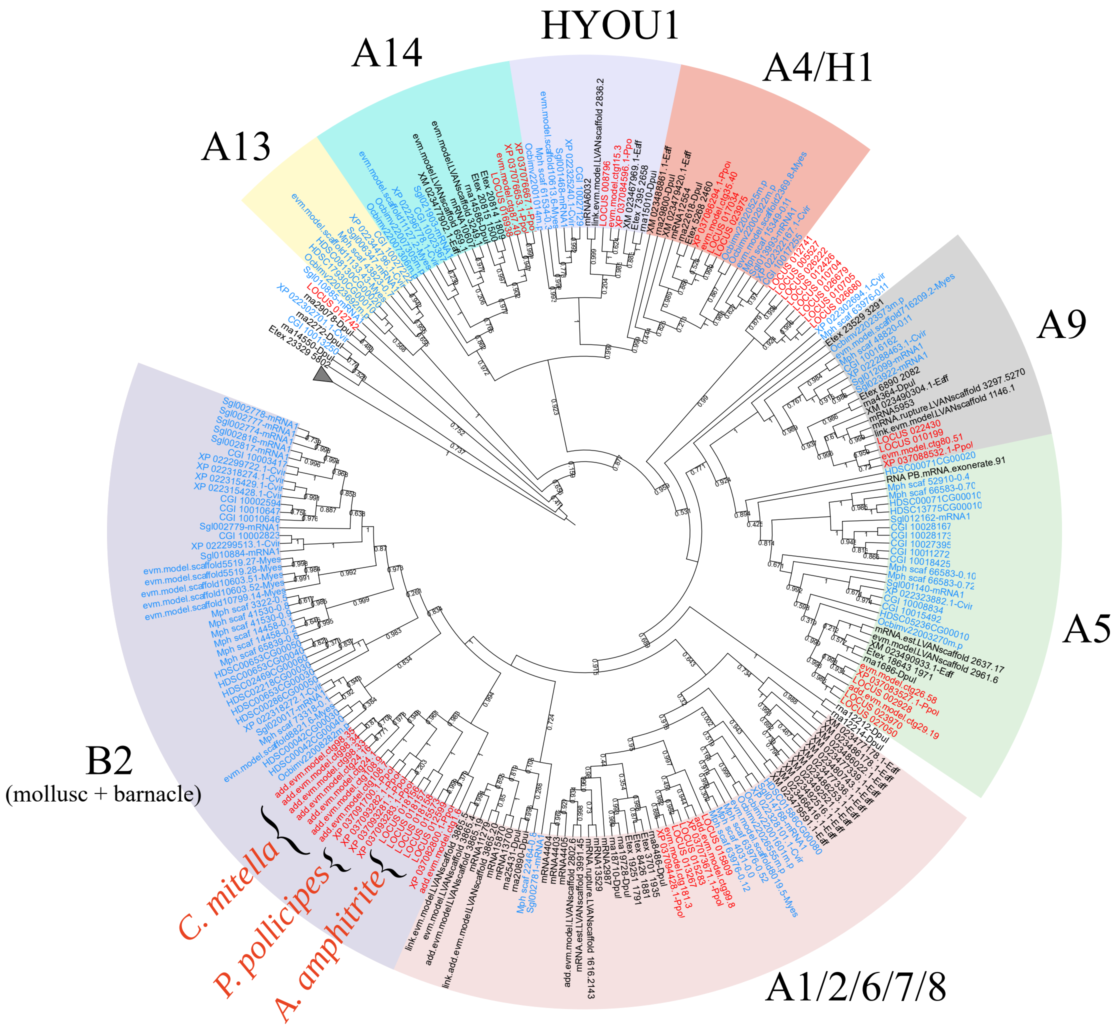

Figure S10. The phylogenetic tree of heat shock protein 70 family. Red, blue and black labels in the phylogenetic tree represent genes of barnacles, molluscs, and non-intertidal crustaceans, respectively. The collapsed clade represents a large HSPA12 subfamily of molluscs.

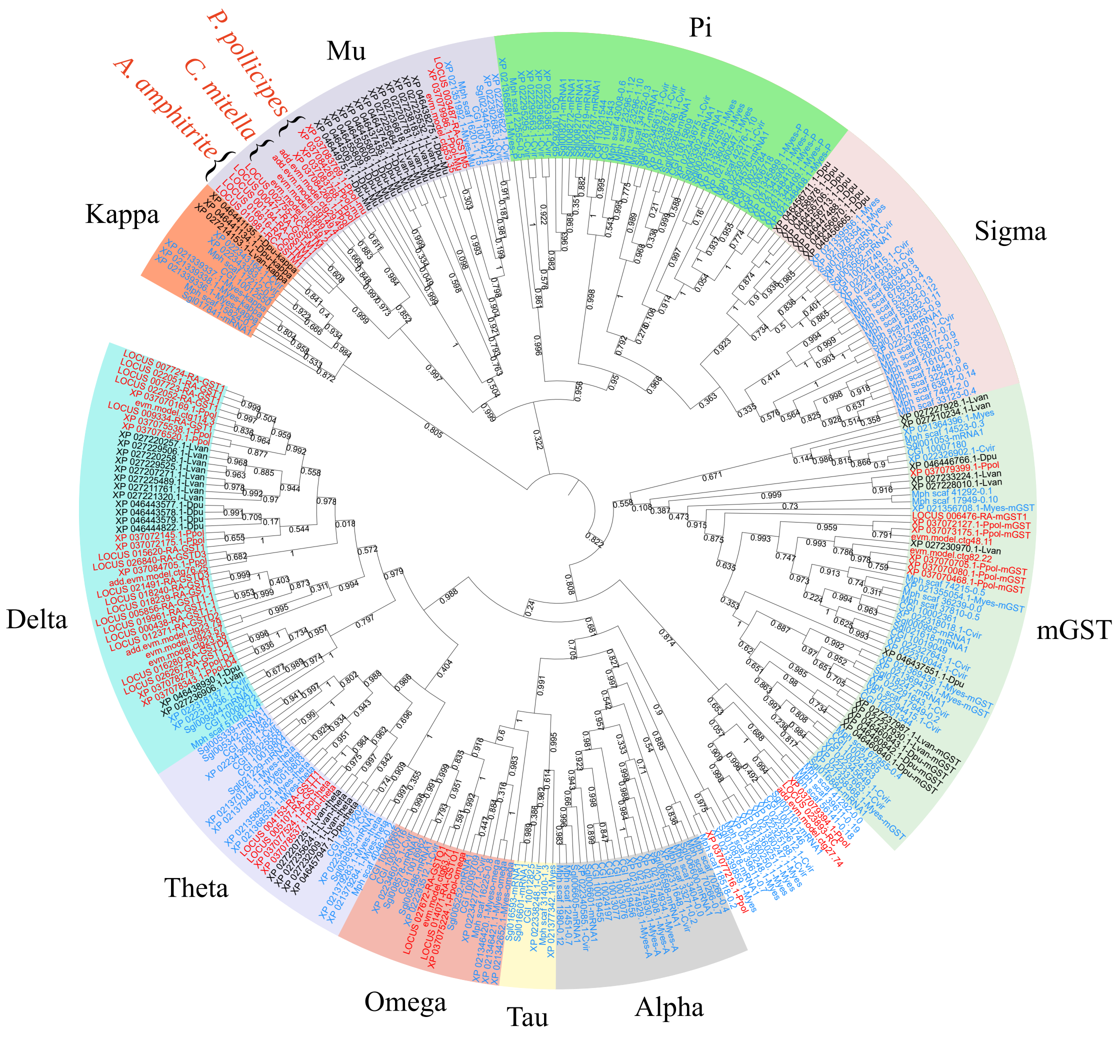

Figure S11. The phylogenetic tree of glutathione S-transferase (GST). Red and blue labels in the phylogenetic tree represent genes of barnacles and intertidal molluscs, respectively. GST Mu were specifically expanded in the barnacle genomes.

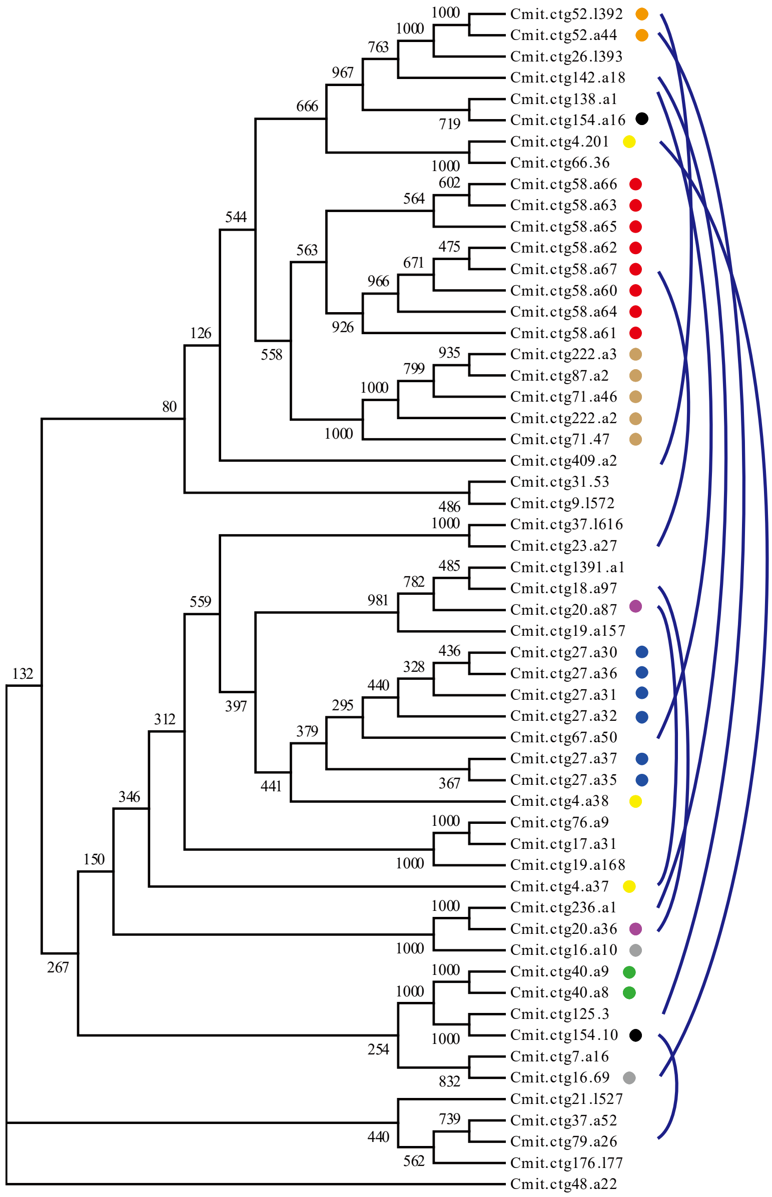

Figure S12. The phylogenetic tree of cytochrome P450 (CYP450) family. Filled circles with different color indicate tandem duplicated CYP450 genes in the *C. mitella* genome. Blue line links two genes indicates the correspondent two genes are ohnologs in syntenic blocks.

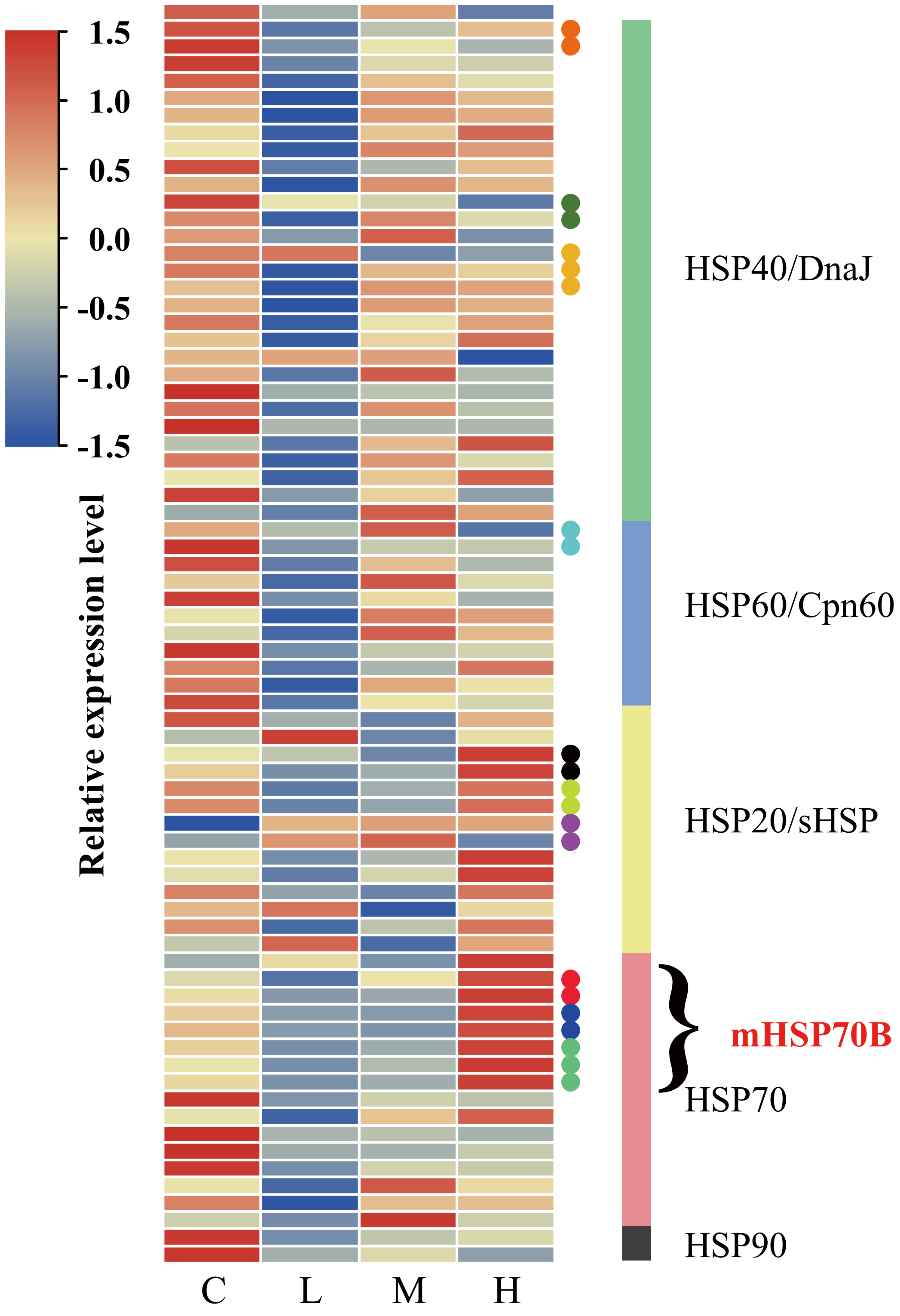

Figure S13. The expression patterns of heat shock proteins of *C. mitella* during air exposure. The expression levels are normalized for each gene. C, L, M and H at the bottom represent the samples collected underwater (control) and at low, middle and high sites from the water level, respectively. The same color symbols on the right represent tandemly duplicated genes in the *C. mitella* genome.

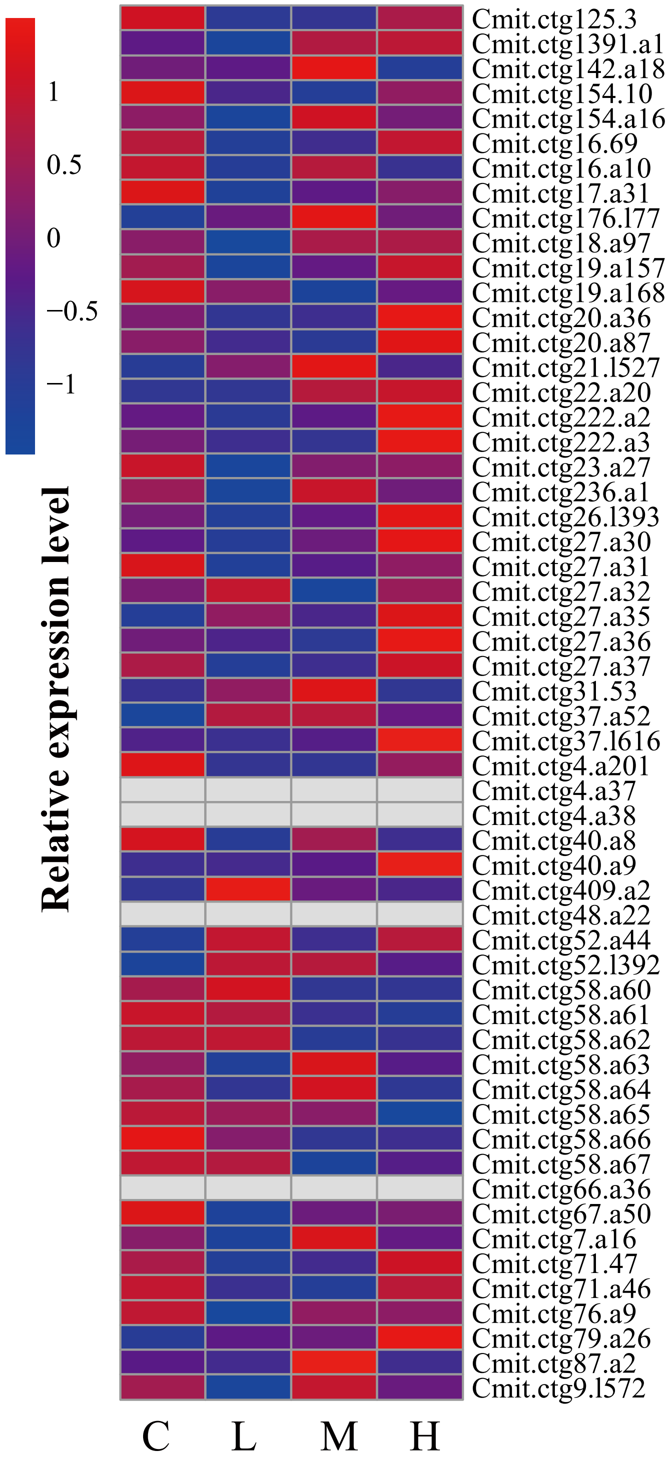

Figure S14. The expression patterns of CYP450s of *C. mitella* during air exposure. The expression levels are normalized for each gene. C, L, M and H at the bottom represent the samples collected underwater (control) and at low, middle and high sites from the water level, respectively.

Supplementary Tables

Table S1. Summary of the genome sequencing clean data of *Capitulum mitella*.*

Sequencing platform	Illumina	PacBio	Hi-C
Size of library	500 bp	20 Kb	500 Kb
Number of Reads	164,836,636 * 2	4,203,896	712110824 * 2
Average length (bp)	150	13,643	100
Total Bases (bp)	49,450,990,800	57,355,949,854	142,422,164,800
Sequencing Depth (X)	~96.28	~111.67	~277.30

* The *C. mitella* genome size was estimated about 513.60 Mb.

Table S2. Summary of the genome assembly of *C. mitella*.

**Primary genome assembly (Contigs)**	
Number	1,959
Total length (bp)	512,057,600
Longest (bp)	17,000,871
N50 (bp)	3,223,513
N90 (bp)	421,594
**Chromosome-level genome assembly (Scaffolds)**	
Number	1,292
Total length (bp)	512,725,600
Longest (bp)	45,597,957
N50 (bp)	30,732,139
N90 (bp)	19,309,488
Chromosome cumber	2n = 32
Anchored contigs (bp)	477,041,753
Anchored contigs (%)	93.04%

Table S3. Summary of the genome assembly of the sequenced crustaceans.

Species	Assembly length	Contig N50	Scaffold N50	Accession No.
Capitulum mitella	512,057,600	3,223,513	30,732,139	This study
Amphibalanus amphitrite	610,566,118	240,779	458,238	PRJNA751628
Armadillidium vulgare	1,717,747,144	38,359	51,088	PRJNA501402
Daphnia pulex	158,607,408	47,461	642,089	PRJNA794129
Eriocheir sinensis	1,293,881,449	26,045	150,053	PRJNA636904
Eulimnadia texana	120,535,642	18,070,303		PRJNA352082
Eurytemora affinis	386,487,637	23,141	252,275	PRJNA423276
Fenneropenaeus chinensis	1,554,960,535	58,996	28,916,617	PRJNA627295
Homarus americanus	2,292,059,586	133,311	759,644	PRJNA655509
Hyalella azteca	596,627,768	5,445	987,977	PRJNA342675
Lepas anserifera	756,396,212	671,058		PRJNA678024
Lepidurus apus	90,320,486	43,471		PRJNA417576
Litopenaeus vannamei	1,618,026,442	57,650	31,296,514	PRJNA438564
Macrobrachium nipponense	1,976,961,095	267,348	83,001,933	PRJNA646023
Parhyale hawaiensis	2,921,140,205	4,003	69,178	PRJNA306836
Penaeus monodon	2,000,783,471	45,084	44,862,054	PRJNA679074
Pollicipes pollicipes	761,780,280	109,725	47,009,503	PRJNA624368
Portunus trituberculatus	1,004,084,517	4,109,061	21,793,880	PRJNA555262
Procambarus virginalis	1,627,847,010	745	39,275	PRJNA356499
Tigriopus californicus	177,715,849	37,992	298,012	PRJNA237968

Table S4. Unigene coverage on the *C. mitella* genome.

Unigenes	Number	Percent
Total unigenes	150,511	100%
Matched unigenes	138,206	91.82%
90% in one scaffold	121,284	80.58%
50% in one scaffold	135,655	90.12%

Table S5. Core gene estimation for *C. mitella* assembly using BUSCO.

	Number	Percentage (%)
Complete BUSCOs	971	91.09
Complete and single-copy BUSCOs	824	77.30
Complete and duplicated BUSCOs	147	13.79
Fragmented BUSCOs	39	3.65
Missing BUSCOs	56	5.25
Total BUSCO groups searched	1066	100

Table S6. Statistics of repeatitive elements for the *C. mitella* genome.

**Species**	***C. mitella***	***A. amphitrite***
Genome length	512,057,600	610,566,118
Repeat length	171236128	268882291
Repeat percent	33.44%	43.83%
SINEs	0.13%	0.00%
LINEs	1.94%	2.98%
LTR elements:	1.59%	1.99%
DNA elements:	5.91%	0.97%
Unclassified:	23.50%	34.00%
Total interspersed repeats:	33.07%	39.93%
Simple repeats:	0.59%	3.54%
Satellites:	0.16%	0.03%
Low complexity:	0.01%	0.46%

Table S7. Statistics of the annotated gene features for *C. mitella*.

**Species**	***Capitulum mitella***
Gene number	13,364
Gene density (gene_number/100kb)	2.61
Gene average length	1,554 bp
Exon number per Gene	7.32
Intron number per Gene	6.32
Exon average length (bp)	212.0 bp
Intron average length (bp)	2137.3
Genome GC percent%	49.93%
Exon GC percent%	67.63%
Intergenic region average length	13,356.5bp

Table S8. KEGG enrichment of the genes retained from whole-genome duplication.

Pathway ID	KEGG class*	Pathway	Pvalue	Qvalue
ko04360	O	Axon guidance	1.72E-12	2.73E-10
ko04010	E	MAPK signaling pathway	5.40E-10	5.71E-08
ko04015	E	Rap1 signaling pathway	2.58E-08	2.01E-06
ko04520	C	Adherens junction	3.18E-08	2.01E-06
ko04014	E	Ras signaling pathway	3.45E-07	1.82E-05
ko04080	E	Neuroactive ligand-receptor interaction	1.12E-06	4.62E-05
ko04514	E	Cell adhesion molecules (CAMs)	1.17E-06	4.62E-05
ko04625	O	C-type lectin receptor signaling pathway	1.33E-06	4.69E-05
ko04910	O	Insulin signaling pathway	2.79E-06	8.08E-05
ko04660	O	T cell receptor signaling pathway	2.80E-06	8.08E-05
ko04390	E	Hippo signaling pathway	1.30E-05	3.44E-04
ko04310	E	Wnt signaling pathway	1.65E-05	3.73E-04
ko04391	E	Hippo signaling pathway -fly	1.81E-05	3.82E-04
ko04361	O	Axon regeneration	2.24E-05	4.45E-04
ko04062	O	Chemokine signaling pathway	5.42E-05	1.01E-03
ko04151	E	PI3K-Akt signaling pathway	0.0001959	3.45E-03
ko04740	O	Olfactory transduction	0.0002742	4.57E-03
ko04810	C	Regulation of actin cytoskeleton	0.0004585	6.61E-03
ko04658	O	Th1 and Th2 cell differentiation	0.0005546	7.32E-03
ko04722	O	Neurotrophin signaling pathway	0.0006453	7.87E-03
ko04728	O	Dopaminergic synapse	0.000679	7.97E-03
ko04150	E	mTOR signaling pathway	0.0008154	8.78E-03
ko04916	O	Melanogenesis	0.0008237	8.78E-03
ko04530	C	Tight junction	0.0008305	8.78E-03

* KEGG classes include organismal systems (O), environmental information processing (E) and cellular processes (C).

Table S9. KEGG enrichment of the differentially expressed genes during air exposure in *C. mitella*.

Pathway ID	Pathway	Qvalue
ko00520	Amino sugar and nucleotide sugar metabolism	9.05E-08
ko04142	Lysosome	3.73E-06
ko04145	Phagosome	1.83E-04
ko04974	Protein digestion and absorption	1.83E-04
ko04972	Pancreatic secretion	2.82E-04
ko04612	Antigen processing and presentation	3.06E-03
ko00061	Fatty acid biosynthesis	5.78E-03
ko00604	Glycosphingolipid biosynthesis - ganglio series	1.59E-02
ko04210	Apoptosis	3.92E-02
ko00531	Glycosaminoglycan degradation	4.94E-02
ko01212	Fatty acid metabolism	4.94E-02
ko04514	Cell adhesion molecules (CAMs)	8.69E-05
ko04610	Complement and coagulation cascades	0.00483
ko04612	Antigen processing and presentation	0.015131
ko04213	Longevity regulating pathway - multiple species	0.028799

Table S10. Summary of gene families involved in intertidal zone adaptation.*

Gene family	Aamp	Cmit	Ppol	Cgig	Cvir	Sglo	Mph	Myes	Obim	Hdis	Tcal	Lvan	Avul
HSP10/Cpn10	2	1	2	1	3	4	1	3	1	0	1	0	1
HSP20	6	14	14	19	19	14	14	6	7	11	15	27	10
HSP40/DnaJ	42	29	37	51	62	46	40	46	34	23	40	30	68
HSP60/Cpn60	19	11	16	16	17	17	15	13	13	10	12	11	11
HSP70	27	16	13	111	172	112	86	63	12	15	14	17	94
HSP90	3	2	2	3	7	4	3	3	5	3	2	5	4
SOD	14	8	13	11	14	8	10	8	4	4	6	3	5
CAT	3	3	3	3	2	2	1	2	1	0	1	1	2
PRX	7	5	7	3	5	4	4	4	4	2	5	5	4
GPx	5	4	3	9	8	6	9	9	4	5	4	10	7
GST delta	13	5	8	1	2	1	1	0	0	1	5	12	4
GST mu	6	5	5	1	2	1	1	1	1	1	2	1	14
P450	88	65	96	133	111	113	234	113	60	108	55	55	165
Tret1	45	26	31	0	0	0	0	0	0	0	31	16	27
FREP	92	25	3	203	158	580	204	60	13	84	17	38	25
IAP	2	1	1	48	22	45	14	13	1	7	4	3	4

*The abbreviation of genes include heat shock protein (HSP), chaperonin (Cpn), chaperone protein DnaJ (DnaJ), superoxide dismutase (SOD); catalase (CAT); peroxiredoxin (PRX), glutathione peroxidase (GPx), glutathione S-transferase (GST), cytochrome P450 (P450), facilitated trehalose transporter 1 (Tret1), fibrinogen-related domain containing protein (FREP), inhibitor of apoptosis protein (IAP). The included species are *Amphibalanus amphitrite* (Aamp), *C. mitella* (Cmit), *Crassostrea gigas* (Cgig), *Crassostrea virginica* (Cvir), *Saccostrea glomerata* (Sglo), *Modiolus philippinarum* (Mphi), *Mizuhopecten yessoensis* (Myes), *Octopus bimaculoides* (Obim), *Tigriopus californicus* (Tcal), *Litopenaeus vannamei* (Lvan) and *Armadillidium vulgare* (Avul).
